# Fully biodegradable hierarchically designed high-performance nanocellulose piezo-arrays

**DOI:** 10.1126/sciadv.ads0778

**Published:** 2025-01-15

**Authors:** Sujoy Kumar Ghosh, Francesca Matino, Fabio Lineu Favrin, Ilaria Tonazzini, Rosarita D’Orsi, Jose Gustavo de la Ossa, Andrea Camposeo, Jun Li, Wenjian Liu, Timothy A. Hacker, Dario Pisignano, Alessandra Operamolla, Xudong Wang, Luana Persano

**Affiliations:** ^1^ NEST, Istituto Nanoscienze-CNR and Scuola Normale Superiore, I-56127 Pisa, Italy.; ^2^ Dipartimento di Fisica “E. Fermi,” Università di Pisa, Largo B. Pontecorvo 3, I-56127 Pisa, Italy.; ^3^ Dipartimento di Chimica e Chimica Industriale, Università di Pisa, via Giuseppe Moruzzi, 13, 56124 Pisa, Italy.; ^4^ Department of Materials Science and Engineering, University of Wisconsin-Madison, Madison, WI 53706, USA.; ^5^ Cellular and Molecular Arrhythmia Research Program, Department of Medicine, University of Wisconsin, Madison, WI 53706, USA.; ^6^ CISUP, Centro per l’Integrazione della Strumentazione dell’Università di Pisa, I-56126 Pisa, Italy.

## Abstract

While piezoelectric sensing and energy-harvesting devices still largely rely on inorganic components, biocompatible and biodegradable piezoelectric materials, such as cellulose nanocrystals, might constitute optimal and sustainable building blocks for a variety of applications in electronics and transient implants. To this aim, however, effective methods are needed to position cellulose nanocrystals in large and high-performance architectures. Here, we report on scalable assemblies of cellulose nanocrystals in multilayered piezoelectric systems with exceptional response, for various application scopes. The submicrometer patterning with effective-flow topography and multilayer stacking promote piezoelectric performance. Record output power and pressure sensitivity in the gentle touch range are obtained in flexible, fully biodegradable systems with stable piezoelectric properties and demonstrated compatibility with different cell lines and implanted devices. These architectures offer new design principles for piezoelectric sustainable materials and for realizing an innovative class of practical components for mechanical energy harvesting and biologically relevant wearables and implants.

## Teaser

Hierarchical nanocellulose arrays offer noninvasive energy solution for self-powered micro-electronics and transient implants.

## INTRODUCTION

Direct piezoelectricity, namely, the generation of electrical polarization in strained materials and the consequent conversion of mechanical energy into electrical energy, has been highlighted in natural materials, such as wood and cellulose, since 1955 (*1*). Piezoelectric effects were later evidenced in several other non-centrosymmetric biopolymers and biological systems, such as bone tissue, proteins, amino acids crystals, and bacteriophages (*2*–*6*). Today, piezo-sensing and energy harvesting device platforms still largely rely on inorganic piezoelectrics, such as titanates and other oxides, and synthetic polymers, such as poly(vinylidene fluoride) (PVDF) and its derivatives (*7*–*10*). However, a few relevant natural biomaterials, including proteins (e.g., silk and collagen), amino acids (e.g., glycine), and polysaccharides (e.g., cellulose and chitin) (*5*, *11*–*13*) are very appealing for their ease of processing and biocompatibility, which can result in remarkable added-value, in particular for biomedical applications. Natural biomaterials might combine piezoelectric properties and biocompatibility with biodegradability and bioresorbability, thus being optimal building blocks of sustainable components in electronics and sensing, as well as of transient implants for health care monitoring and tissue engineering (*14*).

Cellulose, the main structural constituent used in the plant kingdom, is one of the most abundant polysaccharides in nature, and it is highly interesting in this framework (*15*, *16*). It is biodegradable, and methods to extract or texture it at the nanoscale (*17*–*19*) might lead to overcoming issues of conventional cellulosic materials, such as the lack of versatility of predetermined multilength scale hierarchical structures, by enabling the engineering of hydrogen bond patterns (*12*, *20*, *21*) and consequently of piezoelectric features. Cellulose nanocrystals (CNCs) with rod-like shape and high aspect ratio (length of 50 to 500 nm, diameter of about few nanometer) (*19*) are a relevant example. Their films show a notable piezoelectric response (*12*, *22*), and a very high electric dipole moment is reported along the CNC long axis (*23*). Among factors shown to contribute to enhancing piezoelectricity in CNC systems, alignment by electric fields, shear forces, or controlled interfacial interactions are especially effective and easy to modulate (*12*, *22*, *24*). Film casting usually leads CNCs to lay with their length parallel to the substrate plane, i.e., to generate in-plane polarization and shear piezoelectricity (*22*), whereas vertically aligning the longitudinal axis of nanocrystals along the film thickness can result in stronger out-of-plane piezoelectric effects, with measured *d*_33_ coefficients in line with values found for state-of-art synthetic polymers such as PVDF (*12*). Positioning CNCs in more complex, better performing architectures on large areas is difficult, which has so far hindered their use in practical devices for energy harvesting, biologically relevant wearables, and implants.

By submicrometer patterning to promote device performance, here, we report on a scalable method to assemble CNCs in multilayered piezoelectrics for various application scopes over square centimeter scale with exceptional piezoelectric responses. A record generated output power per unit area up to 0.6 μW cm^−2^ and a pressure sensitivity of 4.2 V/kPa in the 0.76 to 4.55 kPa gentle touch range are obtained in flexible, fully biodegradable systems, with stable piezoelectric properties and demonstrated compatibility with cell lines representative of different tissues. A packaged device based on the CNCs arrays is able to produce consistent outputs when implanted on the epicardium of swine hearts.

## RESULTS

**Fig. 1.**
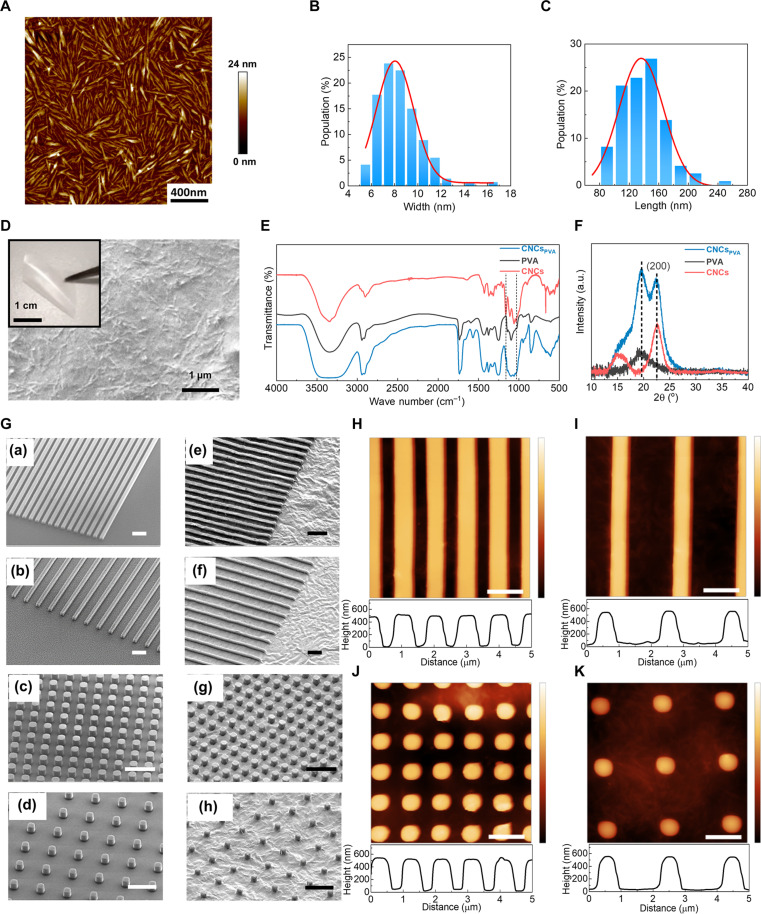
CNCs and patterned CNCs_PVA_ arrays. (**A**) AFM micrograph of CNCs, deposited on a gold-coated silicon substrate from a suspension [0.1% (wt/wt) CNCs/water] and dried overnight at 60°C. (**B**) CNCs width and (**C**) length distributions. The red lines are the fit with a Gauss function to the experimental data. (**D**) SEM micrograph of a film of CNCs_PVA_. Inset: photograph of the film during bending. (**E**) FTIR spectra of freestanding films of CNCs_PVA_, PVA, and CNCs flakes. Spectra are vertically shifted for better clarity. The dashed vertical lines highlight peaks associated to cellulose I at 1161 and 1031 cm^−1^. (**F**) XRD patterns (Cu-Kα1 radiation with wavelength of 1.54056 Å) of CNCs, PVA, and CNCs_PVA_ films on silicon substrates. (**G**) SEM micrographs of the studied patterns [(a) to (d) master templates; (e) to (h) corresponding CNCs_PVA_ structures]. Patterns are shown as follows: [(a) and (e)], NL-1; [(b) and (f)], NL-2; [(c) and (g)], NP-1; and [(d) and (h)], NP-2. Scale bars, 2 μm. (**H** to **K**) AFM planar views and corresponding cross-section profiles of patterned CNCs_PVA_. Vertical scales, 0 to 750 nm. Scale bars, 1 μm.

We prepare CNCs by acid hydrolysis of Avicel PH-101 with 64% aqueous sulfuric acid at 45°C for 80 min. CNCs exhibit average diameter of ~8 nm and length of ~135 nm (Fig. 1, A to C). As shown in the atomic force microscopy (AFM) image in Fig. 1A, they form ultradense networks with complete surface coverage upon casting from water solution. CNCs_PVA_ materials incorporate CNCs in polyvinyalcohol (PVA) upon blending in water and testing different relative concentrations both below and above the percolation (*25*) threshold [0.5 to 3% (wt/wt) CNCs/water and 1 to 20% (wt/wt) PVA/water, respectively], mechanical mixing, spin-coating, and overnight drying at 60°C. CNCs appear evenly dispersed in PVA (0.5 to 22 μm thickness), recapitulating their pristine dense network architecture (Fig. 1D and fig. S1) while retaining flexibility in the resulting freestanding films (inset in Fig. 1D). CNCs interact with PVA through various mechanisms, in particular mobility-suppressing hydrogen bonding, which leads to the formation of birefringent domains (fig. S2) and localized stress patterns without substantially affecting the polymer crystallinity (*26*). Fourier transform infrared spectroscopy (FTIR) measurements performed on CNCs_PVA_ are compared with those of freestanding PVA films and pristine CNCs in Fig. 1E, highlighting characteristic peaks from cellulose I such as those at 1161 cm^−1^ (C─O─C asymmetric stretching at β-glucosidic linkage) and at 1031 cm^−1^ (C─O at C─6 stretching) (*27*). A complex and overlapping H-bonding (*26*) spectral signature is present for all these materials in the region 3000 to 3600 cm^−1^ (magnified in fig. S3). The degree of crystallinity of PVA is stable around 40% for the bare polymer and CNCs_PVA_, as estimated by the intensities of transition peaks at 1144 and 1094 cm^−1^ (*28*). X-ray diffraction (XRD) spectra of CNCs_PVA_ confirm the coexistence of features from cellulose I_β_, i.e., a main peak at 22.5° [(200)] and overlapped secondary peaks at 2θ ≅ 14.8° to 16.5°, and of semicrystalline PVA, with a main diffraction peak at about 20° (Fig. 1F).

CNCs_PVA_ is patterned with submicrometer spatial resolution by a soft-mold imprinting lithography (S-IL) method, through an intermediate, soft polydimethylsiloxane (PDMS) element to transfer the pattern from a silicon master to the final material. Scanning electron microscopy (SEM) images of the master patterns are imaged in Fig. 1G (a to d) and in fig. S4. We use patterns with different feature shape and density, i.e., (a) parallel lines (500 nm width) with 1 μm period (NL-1), (b) the same lines but with 2 μm period (NL-2), (c) pillars (500 nm lateral size) with 1 μm period (NP-1), and (d) the same pillars but with 2 μm period (NP-2). Because of the viscoelastic behavior and plastic flow of the PVA component, by spin-coating on PDMS elements and gentle peeling-off upon drying all the patterns are reproduced with high fidelity in freestanding CNCs_PVA_ as shown in Fig. 1G (e to h) and fig. S5. CNCs might help in increasing the overall mechanical integrity of the flow-guided system during patterning. Minor reductions (<15%) of the lateral size of the transferred features, which do not lead to distortions or pairing, can be ascribed to incomplete filling of the recessed elastomeric features under centrifugal forces, and drying-primed shrinking of CNCs_PVA_ confined in the hydrophobic PDMS (fig. S6). An in-depth AFM study of the surface morphology of nanopatterned CNCs_PVA_ shows well-behaved and regular height profiles, with average height of ~500 nm and full width at half maximum of ~570 nm for the transferred features (Fig. 1, H to K). The surface roughness is 5 to 10 nm on average at the bottom of the features, and as low as 1 to 5 nm at their top. These findings, together with high-magnification SEM micrographs (fig. S7), suggest a relatively lower amount of CNCs reaching the deep recessed features of PDMS during pattern transfer, namely, a vertical gradient of volumetric density of nanocrystals in CNCs_PVA_. Further S-IL experiments, performed on pristine PVA, highlight the smooth appearance of nanopatterned polymeric features (fig. S8). Overall, S-IL is a gentle process that does not lead to changes in structural properties of CNCs as detectable by FTIR and XRD (fig. S9).

**Fig. 2.**
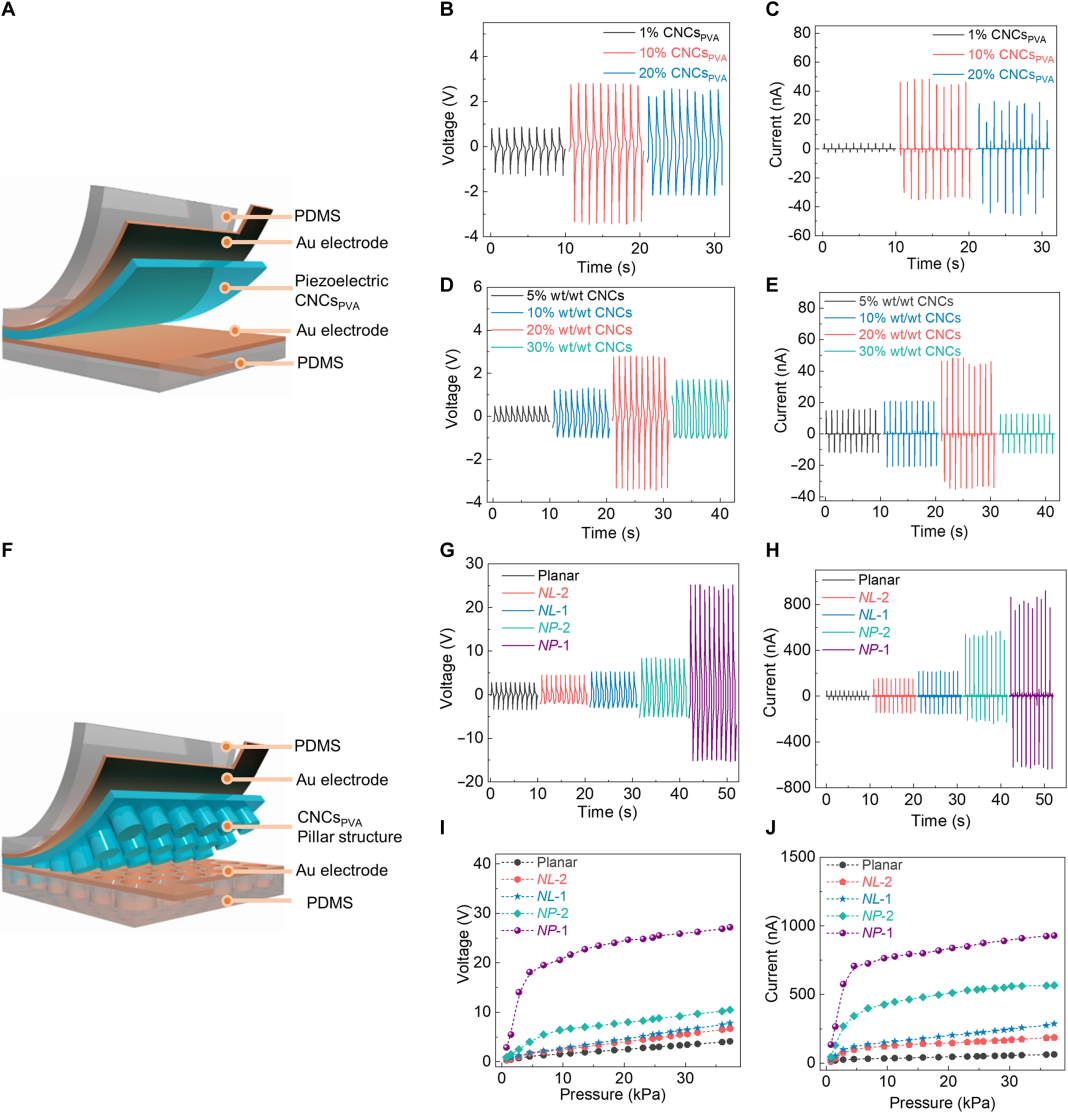
Piezoelectric response of CNCs_PVA_ in MIM architectures. (**A**) Scheme of piezoelectric MIM device based on CNCs_PVA_. (**B** to **E**) Measured voltage [(B) and (D)] and current response [(C) and (E)] of repeatedly compressed (23 kPa) devices obtained by changing the PVA amount [(B) and (C)] at fixed concentration of CNCs in water [2% (wt/wt)], and by changing the relative concentration of CNCs in PVA [(D) and (E)]. Before mixing, the PVA amount is at 10% (wt/wt) in water. (**F**) Scheme of device with nanopatterned CNCs_PVA_, measured voltage (**G**), and current response (**H**) of repeatedly compressed (23 kPa) devices with different transferred patterned. (**I** and **J**) Corresponding pressure response curves.

CNCs_PVA_ is then embedded in metal-insulator-metal (MIM) architectures using 1 × 1 cm^2^ Au electrodes and PDMS as encapsulation layer and poled by applying a 16 kV voltage bias. A scheme of the device is displayed in Fig. 2A, while details of the fabrication process are in Materials and Methods. Poling is carried out by positively biasing a single tip electrode and positioning the sample on a negatively biased copper plate at a distance of a few centimeters from the tip edge. The tip consists of a metallic needle with 0.8 mm diameter and 120 mm length. This process does not affect the XRD diffraction patterns of the CNCs (fig. S10). Different devices with unpatterned CNCs_PVA_ are prepared and tested to define the optimal relative concentration of CNCs and of the polymeric component. As shown in Fig. 2 (B and C) at constant concentration of CNCs in water [2% (wt/wt)], devices obtained with 10% (wt/wt) PVA in water and compressed by a repetitive pressure of 23 kPa generate the highest open-circuit voltage output (*V*_oc_) and short-circuit current (*I*_sc_) (2.8 V and 48 nA, respectively). In these devices, CNCs_PVA_ is 4 μm thick (fig. S11). Lower thickness values leading to higher device capacitance would decrease the voltage output, while at higher thickness the effectiveness of poling is correspondingly reduced (poling field decreased by ~40 times). The thickness dependence of the device output strongly supports the piezoelectric character of the CNCs_PVA_ response (*29*). At the optimal concentration of PVA in water, we then systematically vary the CNCs content [5 to 30% (wt/wt)]. *V*_oc_ and *I*_sc_ outputs increase for relative concentration of the nanocrystals up to 20% in CNCs_PVA_ (from ~0.5 to 2.8 V and from ~15 to 48 nA, respectively). *V*_oc_ and *I*_sc_ outputs then decrease to ~1.7 V and ~13 nA at higher CNC concentration (Fig. 2, D to E), associated with high density of birefringent domains as shown in fig. S2E, flow suppression by higher viscosity (fig. S12) and possibly CNC clustering and reduced film uniformity. Overall, a 20% (wt/wt) optimal CNCs_PVA_ composition is established in terms of piezoelectric behavior, and deployed in S-IL experiments to obtain nanopatterned devices (scheme in Fig. 2F). Figure 2 (G and H) shows the resulting *V*_oc_ and *I*_sc_, respectively, for the different nanopatterned devices under a repetitive pressure of 23 kPa. NP-1 devices show the best performance, exhibiting absolute values *V*_oc_ ~ 25 V and *I*_sc_ ~ 850 nA, which corresponds to an enhancement by about nine (*19*) times of the maximum voltage output (current output) measured in devices with pristine CNCs_PVA_. In addition, the measured pressure-dependent output voltage (Fig. 2I) and current (Fig. 2J) highlight superior pressure sensitivity (voltage sensitivity of 4.2 V/kPa and current sensitivity of 155 nA/kPa, in the pressure range of 0.7 to 5 kPa). *V*_oc_ and *I*_sc_ results are summarized in table S1, while table S2 compares these results with previously published biodegradable and nonbiodegradable piezoelectric devices, as well as triboelectric devices, highlighting the record performance here reported. In addition, we measure the effective piezoelectric coefficients along the poling direction, *d*_33_, for all the patterns, by a quasi-static (Berlincourt) method (*30*). Results (fig. S13) highlight a ~threefold increase of *d*_33_ from planar films to nanopatterned CNCs_PVA_, with the best-performing samples (NP-1) exhibiting *d*_33_ = 35 pC/N. These results are in line with reduced in-plane stress relaxation (parallel to the substrate), with a consequently increase of the strain along the pillar height (*31*, *32*) as well as favored stress transfer of NP-1 topographies to CNCs (*33*), thus generating an enhancement of the voltage output.

**Fig. 3.**
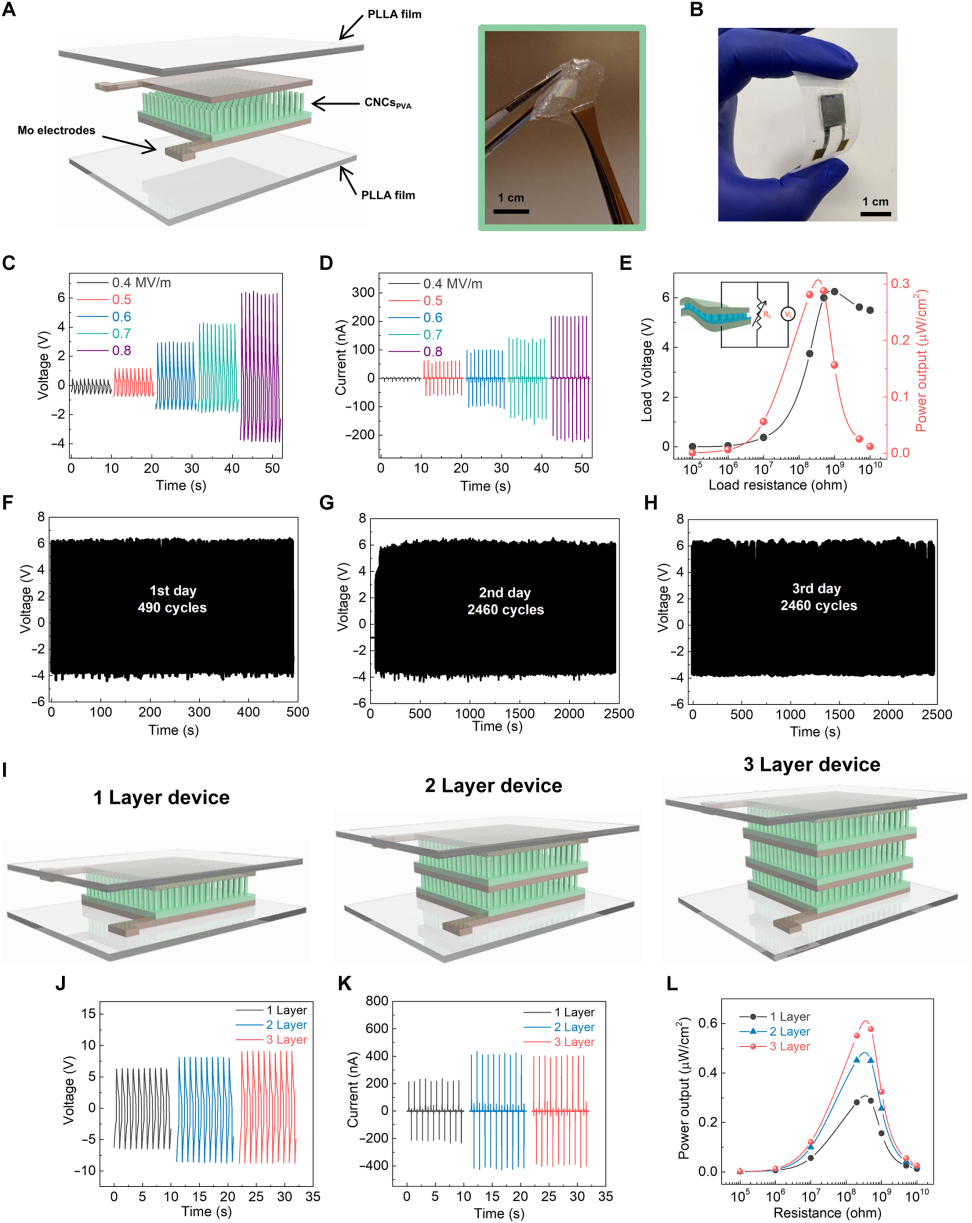
Biodegradable devices with nanopatterned CNCs_PVA_ and their piezoelectric performance. (**A** and **B**) Schematic illustration (A) and photograph (B) of a biodegradable piezoelectric device with nanopatterned CNCs_PVA_. A pristine CNCs_PVA_ film with S-IL pillars is photographed in the inset of (A). (**C** and **D**) Voltage (C) and current (D) response of repeatedly compressed (23 kPa) devices, poled with different electric fields. (**E**) Voltage and power output measured by voltmeters with different resistance. (**F** to **H**) Voltage response as a function of time over 490 (F), 2460 (G), and 2460 (H) compression cycles (23 kPa), performed on the same device at 1 Hz. Measurements are performed in three consecutive days. (**I** to **K**) Schemes of biodegradable devices with multilayered architectures (I) and corresponding voltage (J) and current (K) response (poling field of each layer, 0.8 MV/m; 23 kPa pressure cycles at 1 Hz). (**L**) Power output, measured by voltmeters with different resistance.

NP-1 CNCs_PVA_ is then sandwiched between 25-μm-thick molybdenum (Mo) foils to obtain biodegradable piezoelectric devices (process in fig. S14). Figure S15 shows SEM cross-sectional views of on-Mo laminated NP-1 CNCs_PVA_. Further, we encapsulate the Mo-CNCs_PVA_-Mo system in commercially available decomposable polylactic acid (PLA) foils by thermal sealing. The overall architecture is schematized in Fig. 3A, and a photograph of the flexible device is shown in Fig. 3B. The piezoelectric output improves upon increasing the poling electric-field in the range 0.4 to 0.8 MV/m (Fig. 3, C and D), attributable to enhanced dipole alignment in CNCs_PVA_. Larger poling fields may instead damage the CNCs_PVA_ layer, finally resulting in short-circuited devices. For NP-1 CNCs_PVA_ poled by 0.8 MV/m, we measure *V*_oc_ and *I*_sc_ of about 6 V and 200 nA, respectively, under a repetitive pressure of 23 kPa. This system generates a maximum instantaneous output power density of ~0.3 μW/cm^2^, as estimated by $P=\frac{V_{R}^{2}}{R}$, where $V_{R}$ is the output voltage measured by a voltmeter with the resistance, R=500 megohms (Fig. 3E). In addition, the voltage output is highly stable, as shown in Fig. 3 (F to H) with no substantial fatigue measured over 5400 compression cycles at 1 Hz. The embedded S-IL pattern is robust as well, as found by imaging after use (SEM micrograph in fig. S16 and movie S1). These features allow one to use the biodegradable piezoelectric devices for a variety of demonstrations involving consumer electronic components. For instance, a single device can power arrays of several blue LEDs (fig. S17, A and B) or charge different capacitors (fig. S17, C and D). Using medical adhesive bandage, the device is able to detect the subtle pressure of radial artery pulses when applied to the wrist of a 32-year-old male volunteer (fig. S18 and movie S2).

In addition, energy harvesting can be further enhanced by realizing multilayered architectures, as those schematized in Fig. 3I. In this way, NP-1 CNCs_PVA_ poled by 0.8 MV/m and arranged in three layers reaches *V*_oc_ and *I*_sc_ of about 9 V and 400 nA, respectively, measured under a repetitive pressure of 23 kPa (Fig. 3, J and K). The maximum instantaneous output power density is 0.6 μW/cm^2^ (Fig. 3L). Overall, the piezoelectric performance of NP-1 CNCs_PVA_ devices is outstanding compared with previously reported biomaterials-based platforms (table S3). Last, these architectures can also work under cycling bending, in air as well as in liquid environment upon immersion in water (fig. S19, A to D). Figure S19E reports on the device stability, investigated after repeated bending in water environment for several days.

**Fig. 4.**
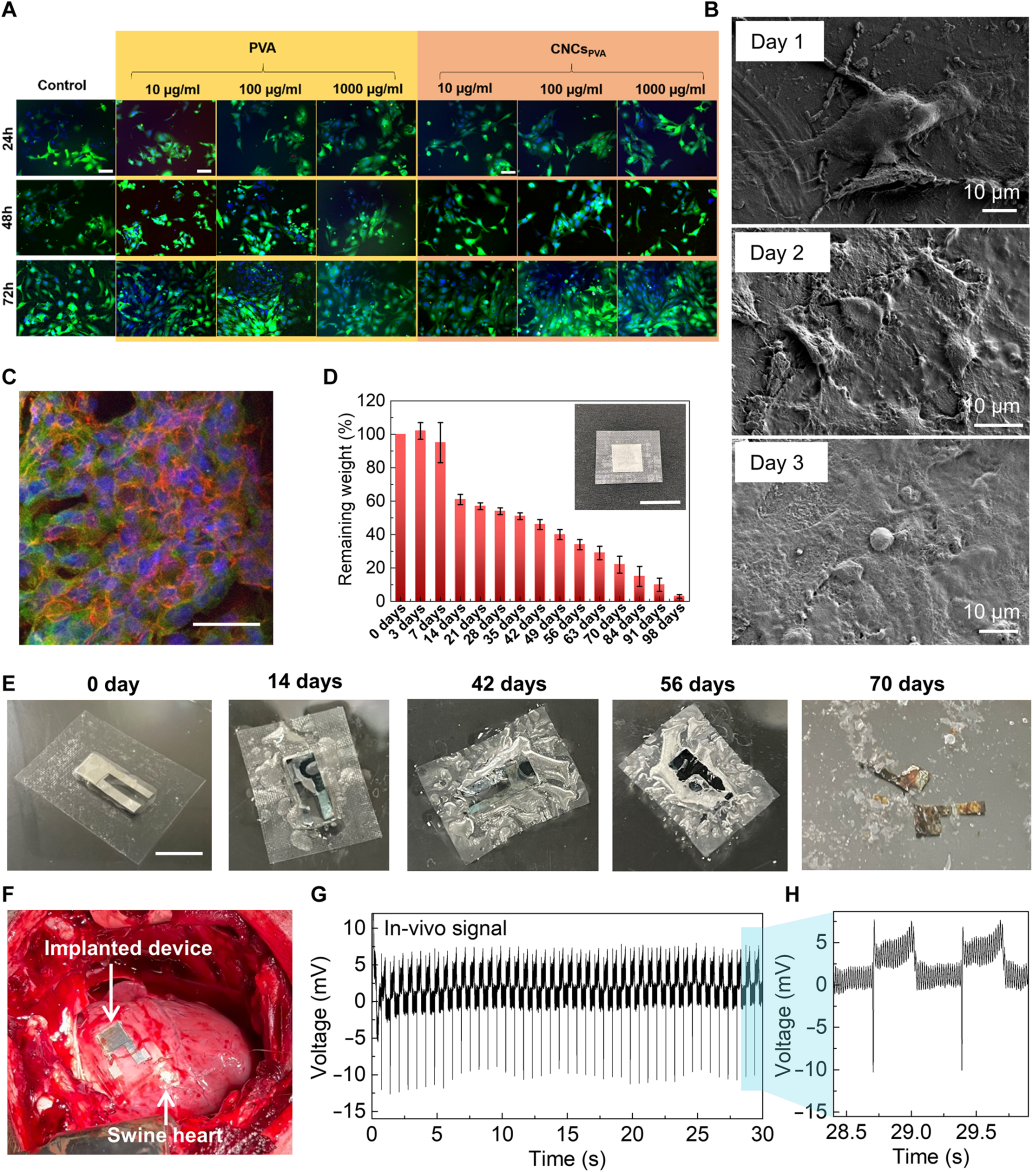
Biocompatibility and implanted devices. (**A**) Vitality test: Fluorescence micrographs of HL-1 cells cultured with various amounts (10, 100, and 1000 μg/ml) of PVA (left) and CNCs_PVA_ (right) dissolved from films in the culture medium during a period of 3 days of observation. HL-1 vital cells are visible in green (calcein positive), all cell nuclei in blue (Hoechst positive), and the level of necrotic/dying cells in red (PI positive). Scale bars, 100 μm. (**B**) SEM micrographs of HL-1 cells cultured on the biodegradable piezoelectric device after 1 to 3 days. (**C**) Representative confocal micrograph of HL-1 cells cultured for 3 days on the CNCs_PVA_ device and immunostained for nuclei (blue), tubulin (green), and actin fibers (red); scale bar, 25 μm. (**D**) Accelerated bioresorbability test at 74°C in PBS solution, for *N* = 10 samples. The remaining material is calculated as the ratio of the dried weight (measured after washing the device in distilled water to remove residual PBS) to the original one (before soaking in PBS). An initial weight increase (about 1% in the first 3 days) might be due to residual water uptake occurring before degradation starts. Inset: photograph of a device with simplified design (smaller area and squared electrodes), used for bioresorbability tests. Scale bar, 1 cm. (**E**) Optical images showing the biodegradable device in the buffered solution at 37°C with stirring at 100 rpm. Scale bar, 2 cm. (**F**) Photograph of the biodegradable device on the right ventricle of an adult swine heart. (**G** and **H**) In vivo recorded voltage response of the device in (F) as a function of time.

Biocompatibility is assessed by in vitro cultures with two cell lines: HL-1 cardiomyocyte cells and A549 lung epithelial cells. CNCs_PVA_ is partially dissolvable in water; hence, cell viability experiments are carried out in the cell medium, supplemented with varied amounts of piezoelectric material (*13*). We use cells cultured in standard media without CNCs_PVA_ or with PVA only as controls, and perform live/dead cell assays by using the green-fluorescent vital dye calcein and propidium iodide (PI), a red-fluorescent dye not permeant to viable cells. As shown in Fig. 4A, HL-1 cells cultured for 24, 48, and 72 hours in the presence of standard, PVA, or CNCs_PVA_ medium are almost all vital (i.e., green) and exhibit a well-behaved spreading and growth with the higher density (i.e., confluent) reached at 72 hours after seeding. We also rule out eventual detrimental effects of the piezoelectric material on cell proliferation (fig. S20A). In vitro studies of HL-1 cells on PVA-CNC devices with Mo electrodes are additionally performed. SEM micrographs of HL-1 cells (Fig. 4B) cultured on the devices at 24, 48, and 72 hours are indicative of a progressively increased area covered by cells on the device surface, finally leading to cell confluence at 72 hours. HL-1 cell proliferation on the devices is also measured. Data in fig. S20B show a small reduction of the number of cells on the device with respect to standard plates at 24 hours, which is recovered already after 48 hours. Because of the central role of cytoskeleton fibers in cell spreading and growth mechanisms, we further investigate the cytoskeleton organization of HL-1 cells grown on the device, by immunolabeling and confocal microscopy (Fig. 4C). Seventy-two hours after seeding, HL-1 cells are fully spread on the biodegradable devices and show organized actin filaments at the cell periphery. Similar results of well-preserved viability and growth are obtained for A549 lung epithelial cells (fig. S21). Together, these data suggest for CNCs_PVA_ and devices based on them a good biocompatibility and safety profile in vitro. Furthermore, we carry out bioresorbability tests by dipping a device with simplified design (photograph in the inset of Fig. 4D) in phosphate-buffered saline (PBS; pH 7.4 at 37°C) up to 112 days. We wash the specimens in distilled water to remove residual PBS, drying and measuring the dried weights. The percentage of the remaining material is estimated as the ratio of the dried weight to the original one (fig. S22). We additionally perform accelerated bioresorbability tests (*34*) at 74°C to highlight faster changes in the weight of the devices immersed in the PBS solution (*35*). Data reported in Fig. 4D highlight a strong reduction of the remaining weight after 14 days of observation, while photographs reported in fig. S23 show that only fragments of Mo remain visible after 100 days. The device behavior upon immersion in the buffer solution at 37°C is additionally captured at different times and reported in Fig. 4E.

Last, to evidence its potential for in vivo application, we applied the device onto the epicardium of swine hearts, assessing its performance during the implantation. The device with a size of 30 × 40 mm^2^ is conformally implanted on the right ventricle of heart by sutures by mini-thoracotomy (Fig. 4F). Driven by heartbeats, the conformally attached device generates peak-to-peak voltages of ~18 mV at a frequency of 1.6 Hz (Fig. 4G), which validates the in vivo biomechanical energy harvesting capability. Moreover, the frequency of device output exactly matches the recorded heart rate of 96 beats/min by a veterinary monitor system, suggesting a perfect synchronization of electricity output and heart movement. The zoomed-in view of voltage outputs in Fig. 4H reveals that each signal consists of a sharp downward peak and a broad upward one, which corresponds to the contraction and relaxation pattern of swine heart. Therefore, the device can also potentially function as a self-powered cardiac sensing system to real-time track the dynamic changes of cardiac systems with information reflected by the electrical signal variations. Moreover, electrocardiograms (ECGs) of swine embedded with the device are collected at the same time (fig. S24). While the ECG provides a normal pattern, no detectable change in the signal patterns between prior implantation and after implantation are observed, suggesting the minimal influences of device on the cardiac function. The interference of device output to heart electrical activity is thus well prevented by the robust PLA package layer.

## DISCUSSION

A method is developed to achieve nanopatterned CNC_sPVA_ arrays in biocompatible and biodegradable device architectures with outstanding piezoelectric performance. At variance with other reported approaches, polymer-embedded nanocellulose undergoes viscous flow shaping leading to different topographies and highly controlled spatial resolution, which lead to multilayer stacking capability and enhanced piezo-response and power output density. Further, compared to other biodegradable force sensors or electrical stimulators (*34*, *36*), this CNC system does not need postprocess steps such as sample cutting to detect normal out-of-plane stress. The platform can be successfully applied for energy harvesting and piezo-sensing both in vitro and in vivo. The reported results provide an effective route for the innovative design and for the realization of scalable, high-performance components based on piezoelectric biomaterials and exploitable in a wide range of systems, including transient implants. Practical applications could be numerous and include components delivering localized electrical cues for cartilage healing (*37*) and regenerative medicine (*38*), transducers for intelligent drug delivery (*39*), and organ monitoring (*40*).

## MATERIALS AND METHODS

### CNCs and CNCs_PVA_ preparation

CNCs are prepared by sulfuric acid hydrolysis. Forty milliliters of deionized water are introduced in a 250-ml three-necked round-bottomed flask equipped with a water condenser and a mechanical stirrer. Then, the flask is cooled in an ice bath, and 40 ml of concentrated H_2_SO_4_ are added. Four grams of Avicel PH-101 are added, and the suspension is warmed to 45°C for 80 min. The system is cooled in an ice bath and 100 ml of deionized water is introduced. The mixture is transferred to polypropylene centrifugation tubes. Centrifugation at 4000 rpm for 10 min is repeated, replacing the supernatant liquid with fresh deionized water until the pH is approximately 1. Then, the precipitate is suspended in deionized water and dialyzed against distilled water until neutrality using a cellulose nitrate membrane with a molecular weight cutoff of 12,400 Da. The resulting suspension is fibrillated with the aid of a sonifier equipped with an ultrasonic horn with a 3.5 mm diameter (microtip) operated in pulsed mode, with a power of 60 W, 0.8 s pulses for 10 min, and then transferred to polypropylene centrifugation tubes and centrifuged at 4000 rpm for 10 min. The supernatant suspension is kept, and water is lyophilized, yielding 2.7 g of CNCs. To obtain CNCs_PVA_ materials, CNCs are first dispersed in 2 ml of deionized water [0.5 to 3% (wt/wt)] using an ultrasonication bath (37 kHz frequency) for 1 hour and vigorously stirring with a vortex mixer for 5 min. A PVA powder (molecular weight, 13,000 to 23,000, Sigma-Aldrich) is added to the dispersion and stirred overnight at 60°C. The polymer concentration is in the range 1 to 20% (wt/wt) with respect to water. CNCs_PVA_ films are prepared by spin-coating on PDMS substrates at 1000 rpm for 1 min, and overnight drying at 60°C. NL and NP patterns by S-IL are obtained by spin-coating CNCs_PVA_ on the patterned side of PDMS (Sylgard 184) molds, realized by replica molding at 80°C for 1 hour from Si masters made by electron-beam lithography (Nanotypos EE). A 9:1 ratio between the prepolymer and the curing agent is used for replica molding. Freestanding CNCs_PVA_ films are then gently peeled off from the elastomeric elements.

### PDMS encapsulated devices

For device preparation, we preliminarily deposit Ti/Au (5/40 nm) electrodes on the patterned side of PDMS molds by thermal evaporation. The CNCs_PVA_ solution is spin-coated on the Au surface treated by plasma oxygen (30 W, 3 min). After overnight drying, samples are poled at 0.4 to 0.8 MV/m for 1 hour. Top Ti/Au (5/40 nm) electrodes are evaporated on the CNCs_PVA_ film. Top and bottom electrical leads consist in stipes of Al foils, attached to the Au surface by adhesive Kapton tape.

### Biodegradable CNCs_PVA_ devices

The bottom electrode of biodegradable devices consists in a Mo foil with different shapes (size > ~1 cm^2^; thickness, 25 μm; Sigma-Aldrich) laminated on the CNCs_PVA_ film at 60°C with an applied pressure of 1.5 kPa (scheme in fig. S14). The top electrode (Mo foil with the same size and thickness) is placed on the opposite side of the film, after peeling off the PDMS substrate. Last, the device is encapsulated within two sheets (25 μm thick) of PLA (Anhui Jumei Biological Technology Co. Ltd) and sealed by hot pressing for 2 s by using a thermal sealer.

### Materials and device characterization

We perform infrared spectroscopy with a FTIR spectrophotometer (Spectrum 100, Perkin-Elmer Inc.), in transmittance mode. SEM micrographs of films, masters, and devices are acquired with a FEG-SEM Merlin from Zeiss at acceleration voltages of 1 to 10 kV. X-ray diffraction measurements are performed on Si-deposited samples at the “Centro di Cristallografia Strutturale” of the University of Florence, by using the Bruker New D8 DaVinci (Cu-Kα1 = 1.54056 Å, 40 kV, and 40 mA) equipped with a Bruker LYNXEYE-XE detector and a secondary monochromator, at a grazing angle of 1.7. AFM measurements are carried out in Peak Force Tapping mode by using a ScanAsyst-Air probe with a nominal spring constant of 0.4 N m^−1^ (Bruker) on a Bruker Dimension Icon system, equipped with a Nanoscope V controller. The surface roughness is calculated from the root mean square values for areas of 0.3 μm^2^, on top and at the bottom of nanopillars. We measure the voltage and current output from the piezoelectric devices using a Precision Source/Measure Unit (Keysight B2911A). Forces applied to the devices are calibrated by using a Single Point Load Cell (model no. 1004, Tedea Huntleigh). *d*_33_ coefficients are measured by using a PiezoMeter System PM300 (Piezotest Pte Ltd). Informed consent was obtained from the participant in radial artery pulses measurements.

### In vitro experiments

We perform bioresorbability tests by soaking the devices in 1 to 10× PBS at 37°C and at 74°C. The percentage of the remaining material is calculated as the ratio of the dried weight (measured after washing the device in distilled water to remove residual PBS) to the original one (before soaking in PBS). HL-1 cardiomyocyte cells (mouse cardiac muscle cell line, Sigma-Aldrich catalog no. SCC065), provided by V. Lionetti (Scuola Superiore Sant’Anna, Pisa), are cultured in Claycomb medium supplemented with 10% fetal bovine serum (FBS), 2 mM glutamine, penicillin (10 U/ml)–streptomycin (10 mg/ml), and 0.1 mM norepinephrine, as in (*41*). For HL-1 cells, all substrates (plates and devices) are coated with fibronectin (5 μg/ml; Thermo Fisher Scientific, 33010018) in a solution of gelatin 0.02% in H_2_O, for 1 hour at 37°C. A549 lung epithelial cells (human lung cancer cell line, ATCC CCL-185) are cultured in RPMI 1640 medium supplemented with 2 mM l-glutamine, 10% FBS, and 1% penicillin-streptomycin. All cell supplements and solutions are from Thermo Fisher Scientific. The cells are kept in a humidified atmosphere of 5% CO_2_ at 37°C by a standard cell incubator (MCO-38AIC-UV, Sanyo Electric Co. Ltd.). We seed cells on 96-well plates (2500 cells per well for A549; 3000 cells per well for HL-1). The day after, cell culture media are supplemented with different concentrations (10, 100, and 1000 μg/ml) of either PVA or CNCs_PVA_, and cultures observed up to 72 hours. For cell proliferation assays, cells are rinsed twice and assayed at 24, 48, and 72 hours using the RealTime-Glo Cell Viability assay (G9712, Promega), following assay instructions (end point format) (*42*). After incubation, the solution of each well is moved to a new well in a black plate, and luminescence is read with a GloMax DISCOVER microplate reader (Promega). The results are reported as percentages of cells with respect to the untreated cells (control condition). Each condition is tested in triplicate, for at least *n* = 3 independent experiments. In parallel, we carry out cell vitality tests by simultaneous imaging of live and dead cells. Calcein (C3100, Molecular probes, Eugene, OR, USA) is used as vital staining, PI (P4864, Sigma-Aldrich) is used as a necrotic marker, and Hoechst 33342 (H3570, Thermo Fisher Scientific) is used as cell nucleic acid staining. HL-1 and A549 cells treated with PVA and CNCs_PVA_ are incubated with calcein (5 μM), PI (1 μg/ml), and Hoechst 33342 (10 μg/ml) in medium for 5 min. Samples are imaged by using a Nikon Eclipse-Ti inverted wide-field fluorescence microscope equipped with a 20× air Nikon objective (numerical aperture, 0.45; Plan-Fluor), an incubating chamber (Okolab), and a CCD ORCA R2 (Hamamatsu). Cell proliferation data are reported as average values ± the standard error of the mean. Results from control experiments are reported as mean ± SD to show intra-assay variability. Data are statistically analyzed using GraphPad PRISM program, version 5.00 (GraphPad Software, San Diego, CA). One-way analysis of variance (ANOVA) analysis (Dunnett’s test) is used to compare different conditions. Student’s *t* test is used to compare two conditions. Statistical significance refers to *P* < 0.05. HL-1 cells are cultured on CNCs_PVA_ devices inside 48-well plates (5000 cells per well) up to 72 hours. We move the devices to a new well after 3 hours from cell seeding, and tested for cell proliferation at 24, 48, and 72 hours. Last, HL-1 cells are fixed for 15 min in 4% paraformaldehyde in PBS and processed for immunostaining, as previously reported (*43*). In brief, cells are stained with anti-tubulin primary antibody (Sigma Merck, 1:200) in GDB buffer (0.2% BSA, 0.8 M NaCl, 0.5% Triton X-100, and 30 mM phosphate buffer, pH 7.4) containing phalloidin-Alexa647 (Thermo Fisher Scientific A22287; 1:40; to stain actin fibers), overnight at 4°C. Samples are then incubated with appropriate AlexaFluor Plus488–conjugated secondary antibodies (Thermo Fisher Scientific; 1:150) in GDB and mounted using Fluoroshield histology mounting medium with 4′,6-diamidino-2-phenylindole (Sigma-Aldrich, F6182). We acquire confocal images by using a laser scanning confocal microscope TCS SP2 (Leica Microsystems) with a 40× oil objective using two laser lines (488 e 647 nm). The reported confocal image is obtained from a *z* series (stack depth was within 10 μm; steps = 1 μm). The resulting *z* stack is processed by ImageJ software (National Institutes of Health) into a single image using “*z*-project” and “Max intensity” options.

### In vivo experiments

All animal experiments are conducted under a protocol approved by the University of Wisconsin Institutional Animal Care and Use Committee. Domestic adult swine (Duroc, Landrace, Large white, ~30 kg) are sedated with intramuscular Telazol (4 mg/kg) and xylazine (2 mg/kg) and then intubated and ventilated with a respirator and anesthetized with isoflurane (2%) and oxygen. Ventilation is adjusted to maintain blood gases in the physiological range. Animals are monitored continuously for anesthetic state by jaw tension, heart rate, blood pressure, end-tidal CO_2_, and oxygen saturation. A surface ECG is monitored through a veterinary monitor system (BM5VET, BioNet) during the entire procedure. A mini-thorocotomy is then performed to implant CNCs_PVA_ devices between the heart and pericardium with the device being sutured on the epicardium. The voltage outputs of implanted energy harvester are measured by connecting probes from a multimeter (DMM 6500, Keithley) to the leads of the device.

## Supplementary Materials

### The PDF file includes:

Figs. S1 to S24 Tables S1 to S3 Legends for movies S1 and S2 References
