# Supplementary material for: Fully biodegradable hierarchically designed high-performance nanocellulose piezo-arrays
Source: Sci Adv. 2025 Jan 15;11(3):eads0778. doi: 10.1126/sciadv.ads0778 (PMC11734713; doi:10.1126/sciadv.ads0778)
Supplement: Supplementary file 1 — Figs. S1 to S24 Tables S1 to S3 Legends for movies S1 and S2 References [file sciadv.ads0778_sm.pdf]

Science Advances
AAAS

Supplementary Materials for

Fully biodegradable hierarchically designed high-performance nanocellulose piezo-arrays

Sujoy Kumar Ghosh *et al.*

Corresponding author: Luana Persano, luana.persano@nano.cnr.it

Sci. Adv. **11**, eads0778 (2025)
DOI: 10.1126/sciadv.ads0778

The PDF file includes:

Figs. S1 to S24
Tables S1 to S3
Legends for movies S1 and S2
References

Other Supplementary Material for this manuscript includes the following:

Movies S1 and S2

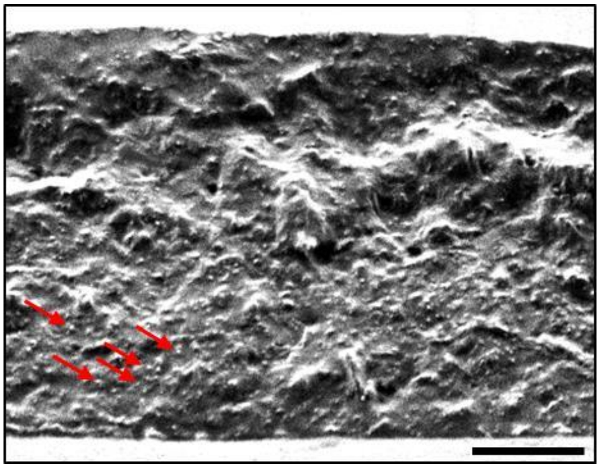

Fig. S1. Dispersion of CNCs in polymer matrix. SEM micrograph of a freeze-fractured $CNCs_{PVA}$ film cross section. Arrows highlight individual nanocrystal-related features across the sample section. Scale bar: 3 μm.

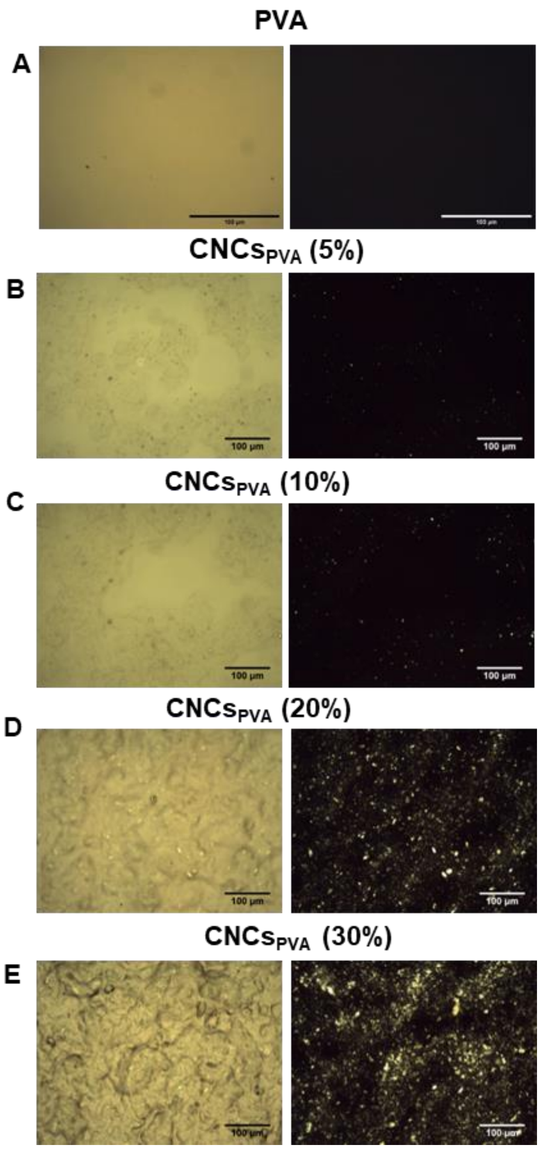

Fig. S2. Optical investigation of polymeric films and $CNCs_{PVA}$ materials. Bright field (left side) and cross-polarized optical micrographs (right side) of films of PVA and $CNCs_{PVA}$ with different amount of the CNC component. The loading content of CNCs in PVA is 0 (A), 5% (B), 10% (C), 20% (D) and 30% (E). Loading contents are expressed as wt/wt relative concentration).

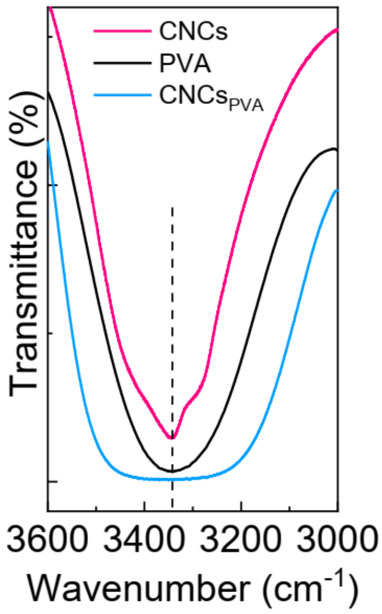

Fig. S3. Fourier-transform infrared spectroscopy (FTIR) investigation highlighting incorporation of CNCs in polymer matrix. FTIR spectra of CNCs$_{PVA}$, PVA and pristine CNCs films (vertically-shifted for better clarity). The vertical dashed line highlights wavenumber values associated to H-bonding of CNCs (3340 cm^{-1}).

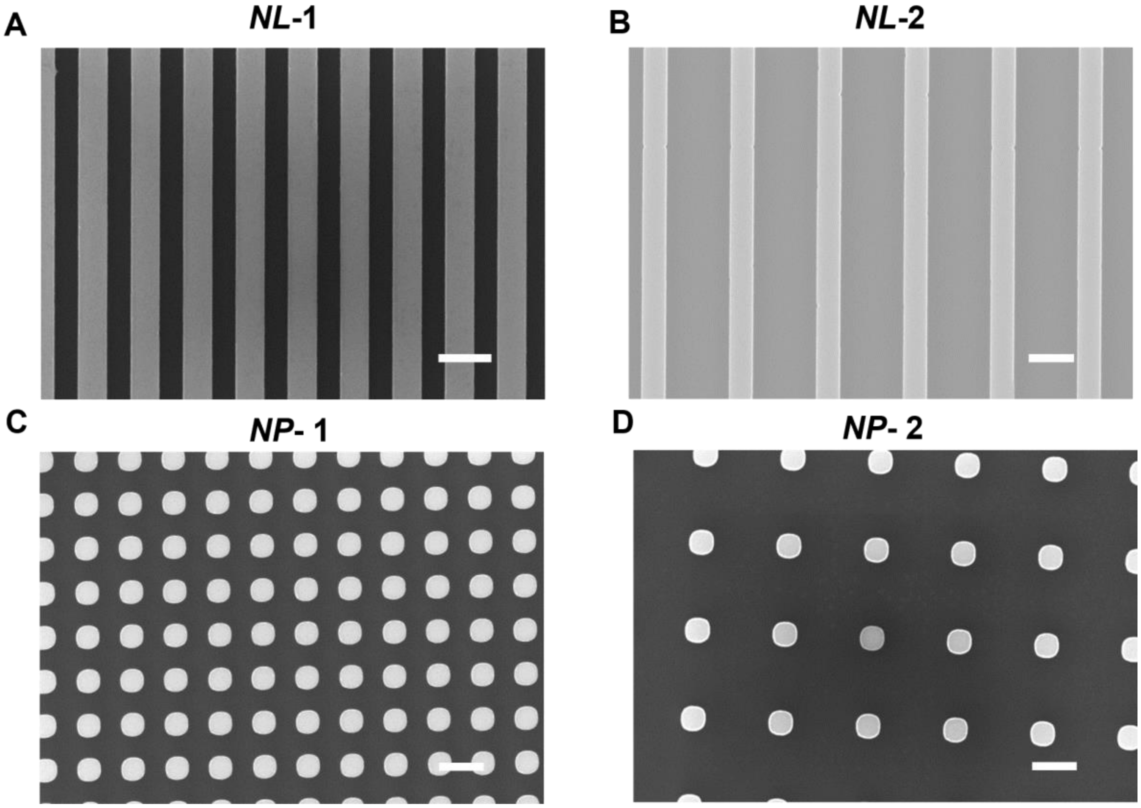

Fig. S4. SEM micrographs of the silicon master used in S-IL experiments, planar views. Patterns are shown as follows: A) *NL*-1, (B) *NL*-2, (C) *NP*-1, (D) *NP*-2. Scale bar: 1 μm.

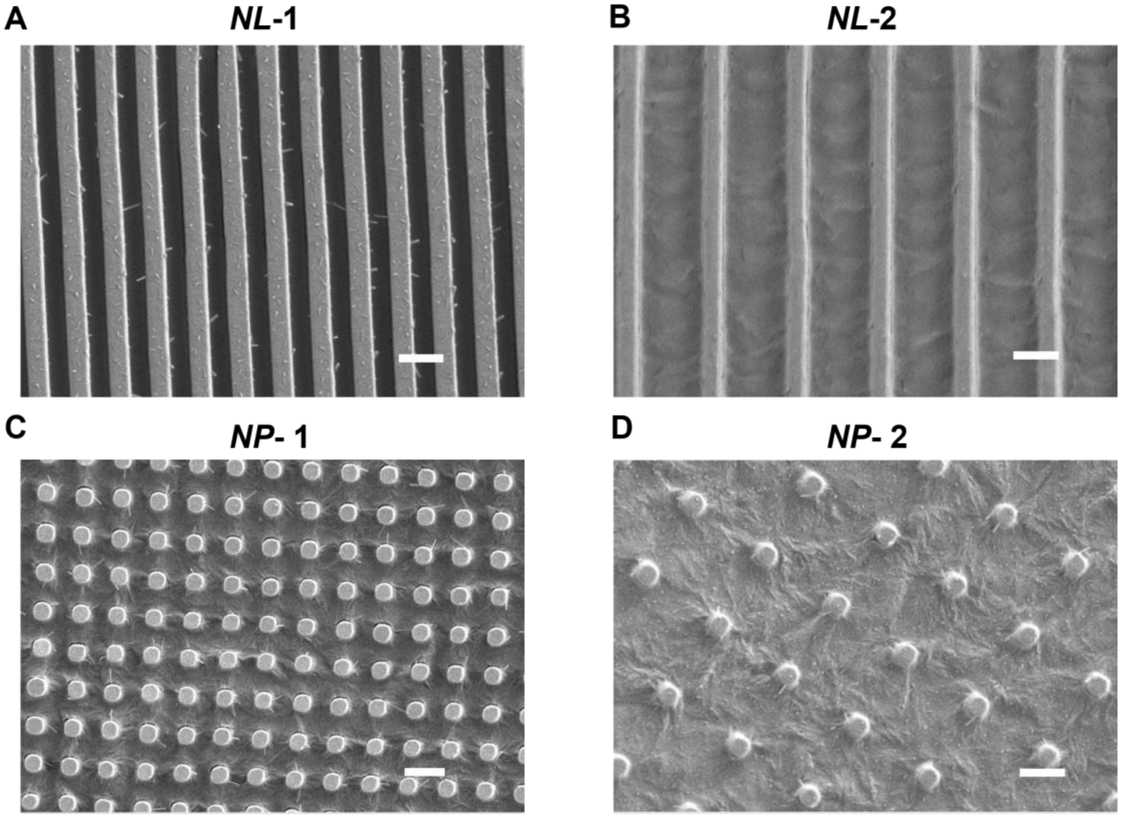

Fig. S5. SEM micrographs of the nanopatterned CNCs$_{PVA}$, planar views. Patterns are shown as follows: A) *NL*-1, (B) *NL*-2, (C) *NP*-1, (D) *NP*-2. Scale bar: 1 μm.

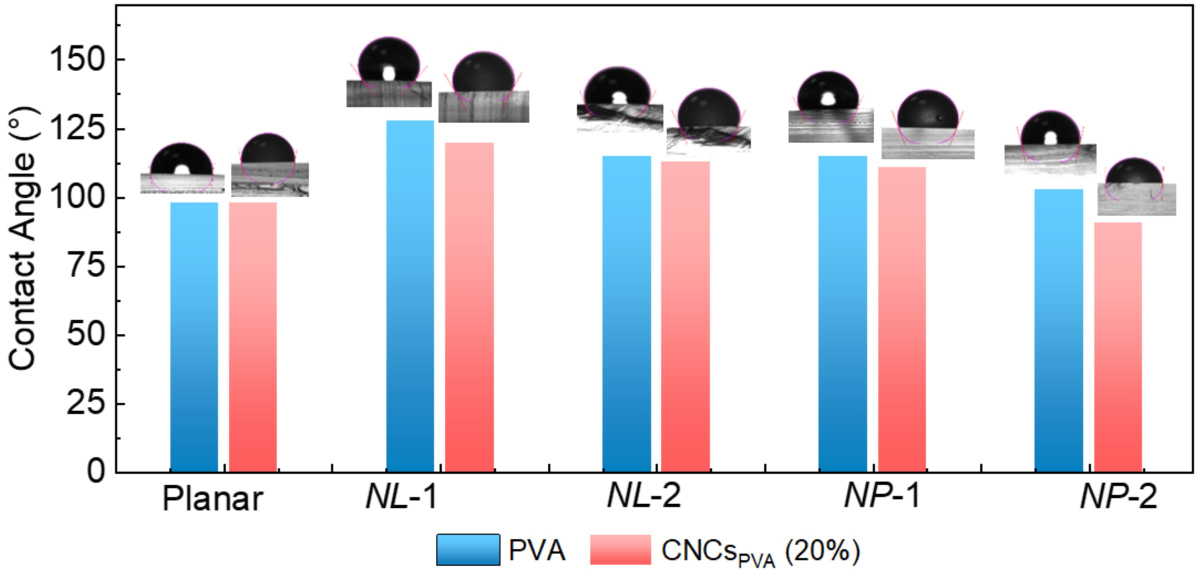

Fig. S6. Wettability properties measured for PDMS surfaces by solutions of PVA and CNCs$_{PVA}$. Measured contact angle values of PVA and CNCs$_{PVA}$ solutions. The corresponding photographs are reported as top insets. The contact angle on the surface of the *NL*-1 and *NL*-2 samples is measured in the direction perpendicular to the length of the lines, where liquid pinning is expected.

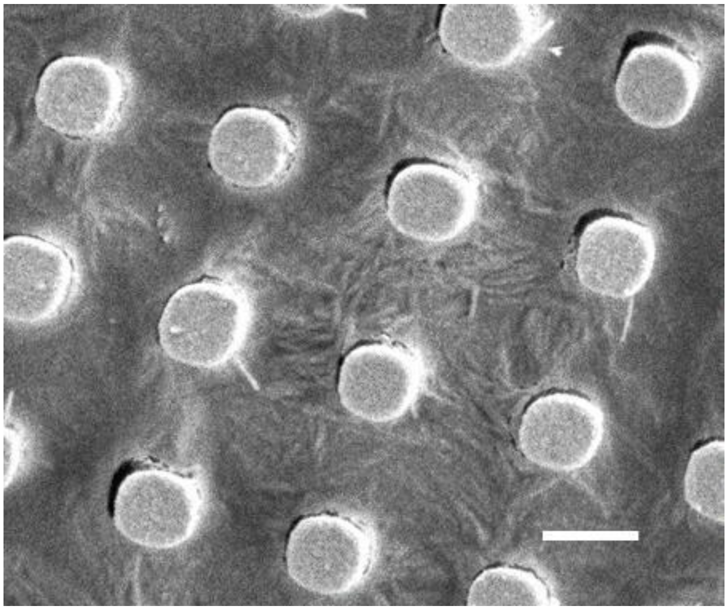

Fig. S7. High-magnification SEM micrograph of *NP*-1 $CNCs_{PVA}$. The presence of CNCs is more clearly visible in the inter-pillar bottom area. Scale bar: 500 nm.

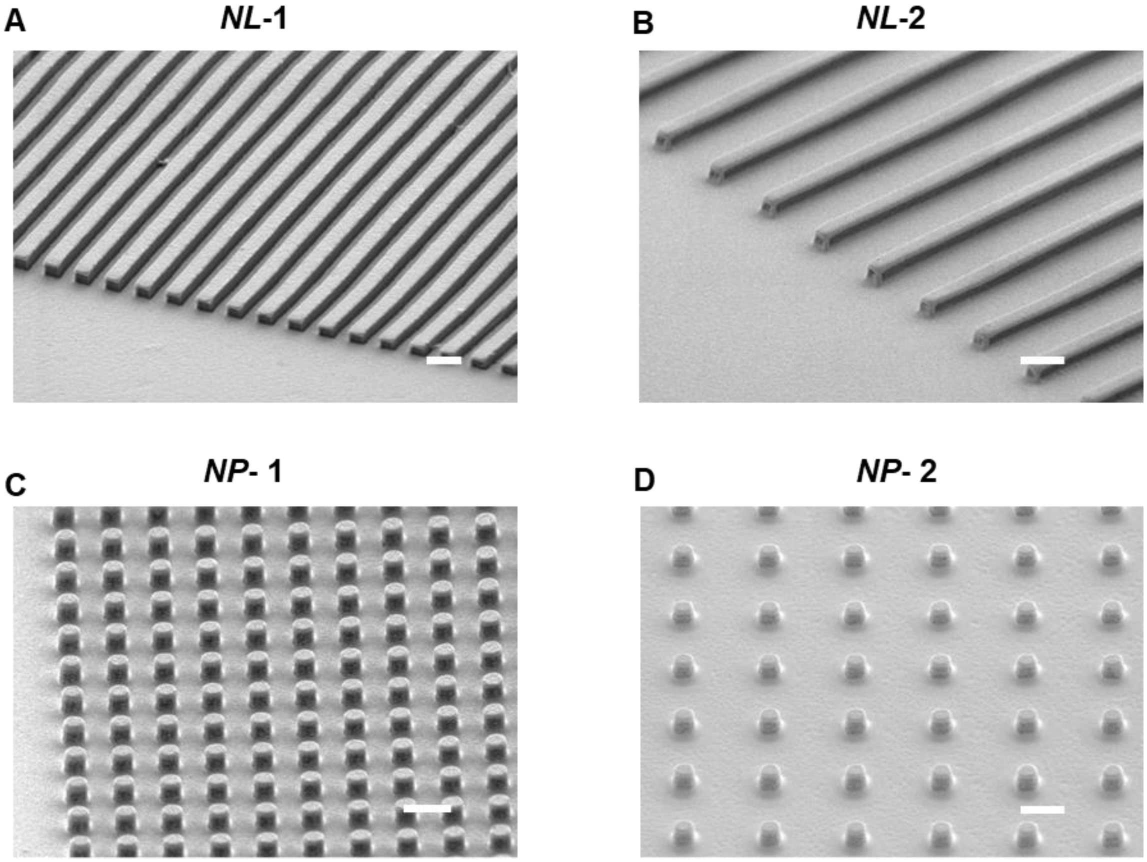

Fig. S8. SEM micrographs show nanopatterned PVA. Patterns are shown as follows:

A) *NL*-1, (B) *NL*-2, (C) *NP*-1, (D) *NP*-2. Scale bar: 1 μm.

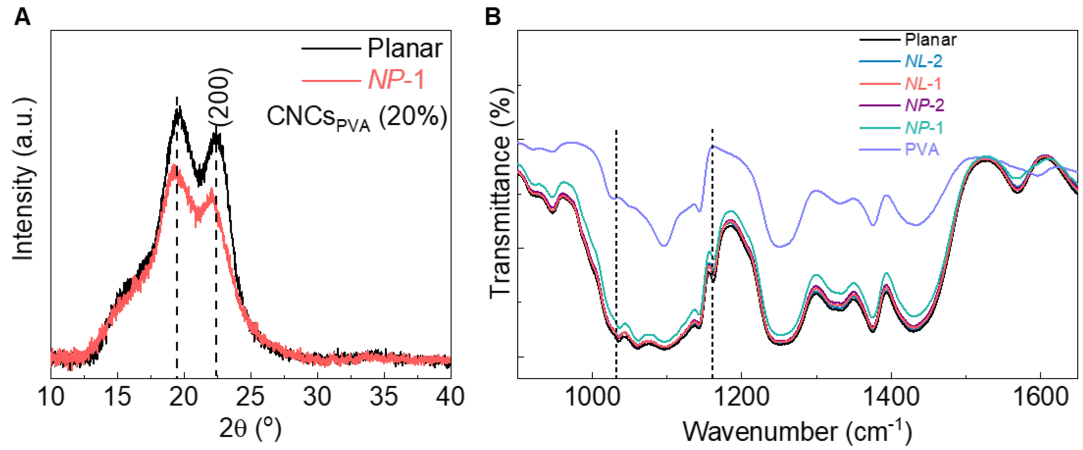

Fig. S9. Nanopatterned CNCs$_{PVA}$ are characterized by XRD and FTIR spectroscopy. A) XRD pattern of pristine (black line) and *NP*-1 (red line) CNCs$_{PVA}$. B) FTIR spectra of pristine PVA and of nanopatterned CNCs$_{PVA}$. The dashed vertical lines highlight peaks associated to Cellulose I at 1161 cm^{-1} and 1031 cm^{-1}. CNCs/PVA relative content = 20% wt/wt.

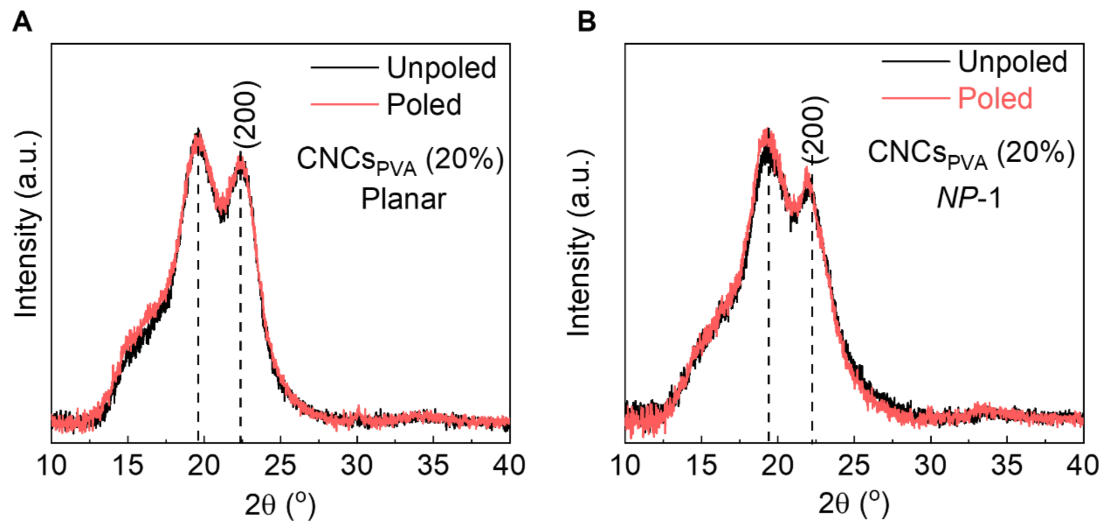

Fig. S10. Either unpoled and poled CNCs$_{PVA}$ characterized by XRD. XRD pattern of unpoled (black line) and poled (red line) (A) planar and (B) *NP*-1 CNCs$_{PVA}$. CNCs/PVA relative content = 20% wt/wt.

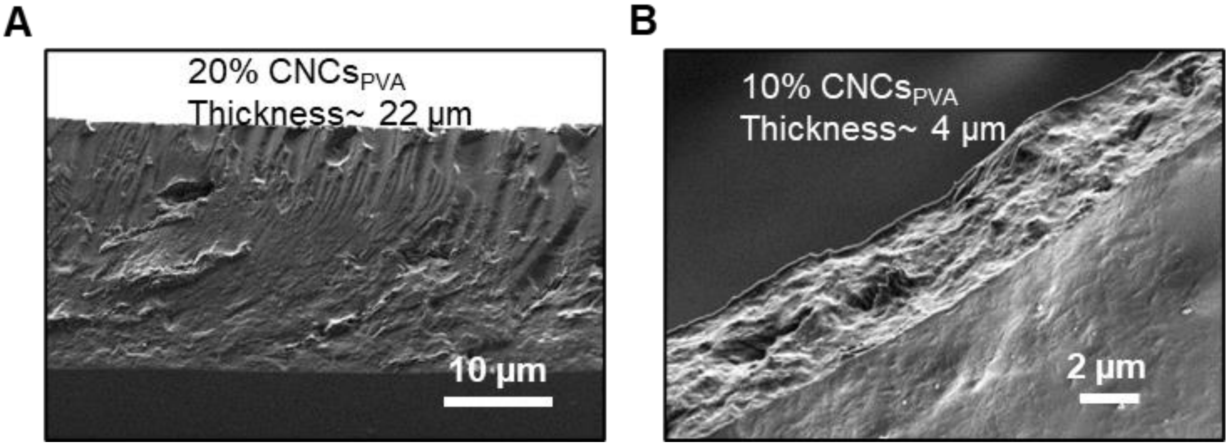

Fig. S11. **CNCs$_{PVA}$ thickness changes with the concentration of PVA in the initial solution.** SEM cross-sectional views of CNCs$_{PVA}$ with different concentrations of PVA in the initial water solution [fixed concentration of CNCs in water (2% wt/wt)]. Concentration value: 20% (A), 10% (B), wt/wt.

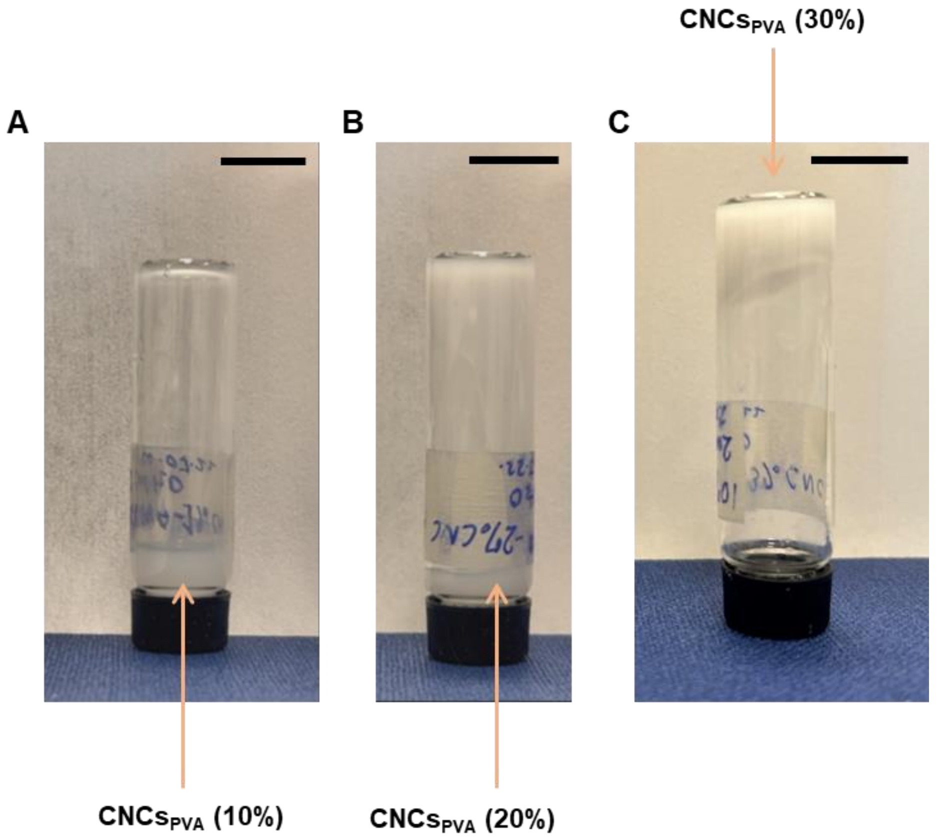

Fig. S12. Photographs of glass vials containing solutions of CNCs$_{PVA}$. Concentration of the CNC component (wt/wt): 10% (A), 20% (B), and 30% (C). After turning the vial upside down, the solutions in (A) and (B) flow down within 1 s, while the solution in (C) does not flow within the first 30 s of observation. Scale bar: 1 cm.

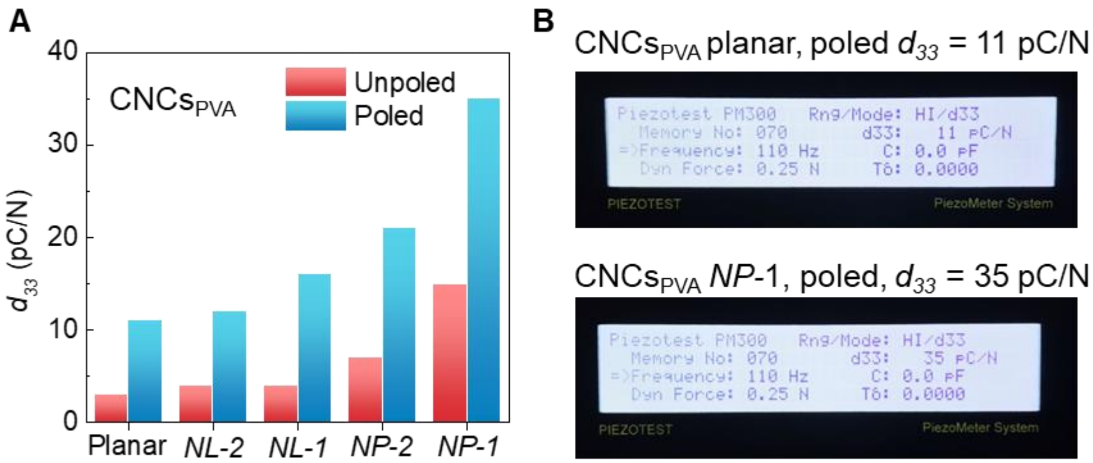

Fig. S13. Piezoelectric coefficients along the poling direction, d_{33}, are measured for all the patterns by a quasi-static method. (A) Measured d_{33} coefficients. (B) Photographs of the display of the d_{33} meter during measurements of planar and *NP*-1 $CNCs_{PVA}$.

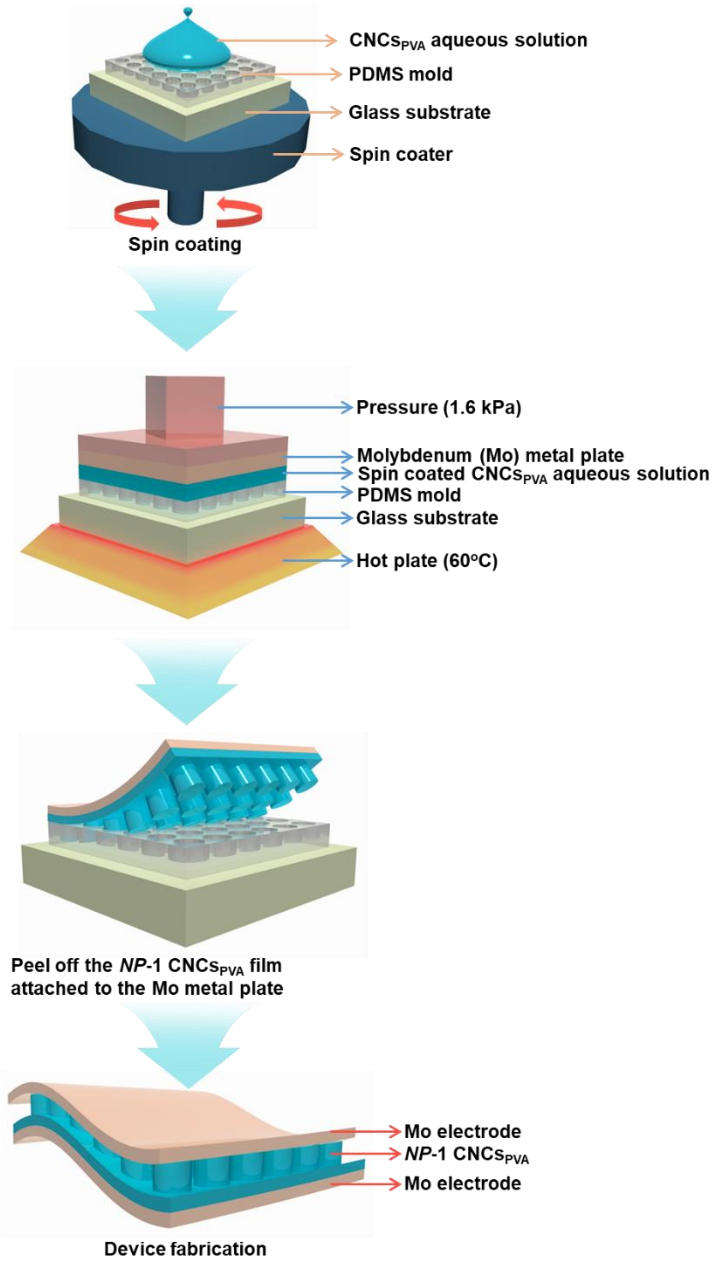

Fig. S14. Scheme of the realization process for biodegradable CNCs$_{PVA}$ devices. *NP*-1 CNCs$_{PVA}$ is sandwiched between 25 μm-thick molybdenum (Mo) foils upon applying pressure to transfer the pattern and peeling off.

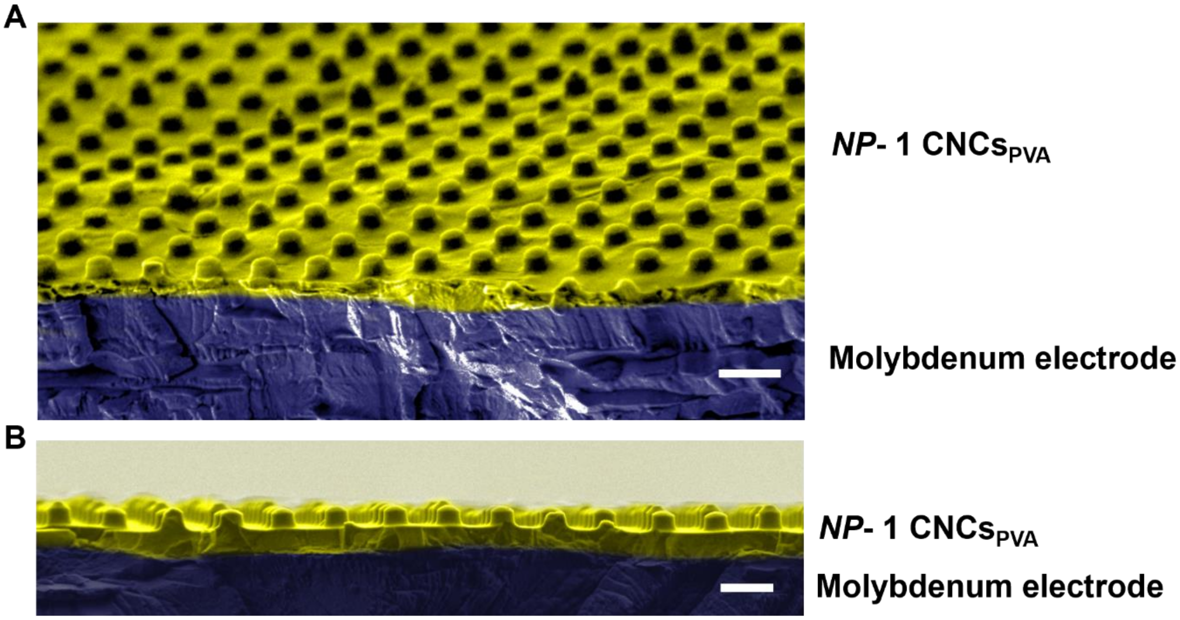

Fig. S15. SEM micrographs show cross-sections of CNCs$_{PVA}$ on Mo. SEM cross-sectional micrographs of *NP*-1 CNCs$_{PVA}$ (yellow) laminated on a Mo electrode (violet). Samples are cleaved and imaged in their cross-section, bird's eye view (A) and angle about 90° (B). Scale bars: 1 μm.

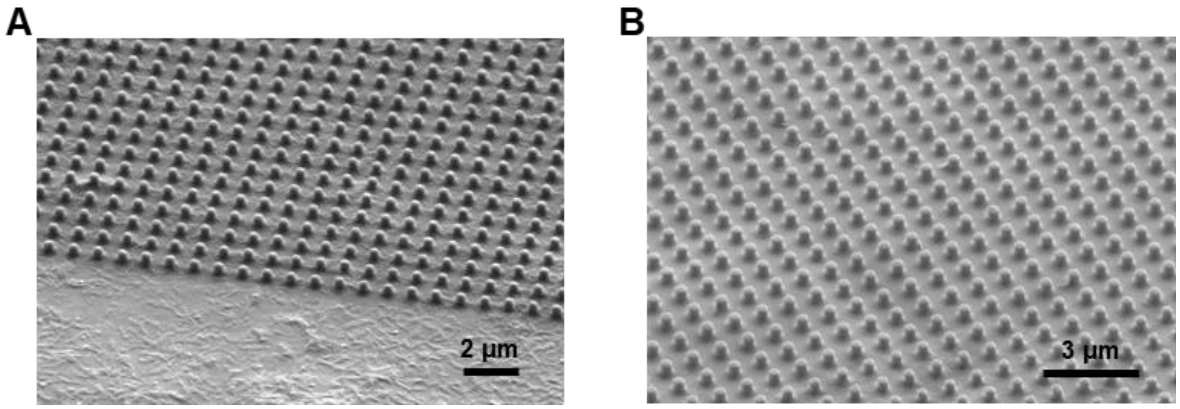

Fig. S16. SEM micrographs evidence pattern robustness. SEM micrographs of *NP*-1 $CNCs_{PVA}$ on Mo electrodes before (A) and after (B) use (repeated 23 kPa compression at 1 Hz, about 15 minutes/days for 7 days). The micrograph in B) is acquired upon removing the top Mo electrode from the device.

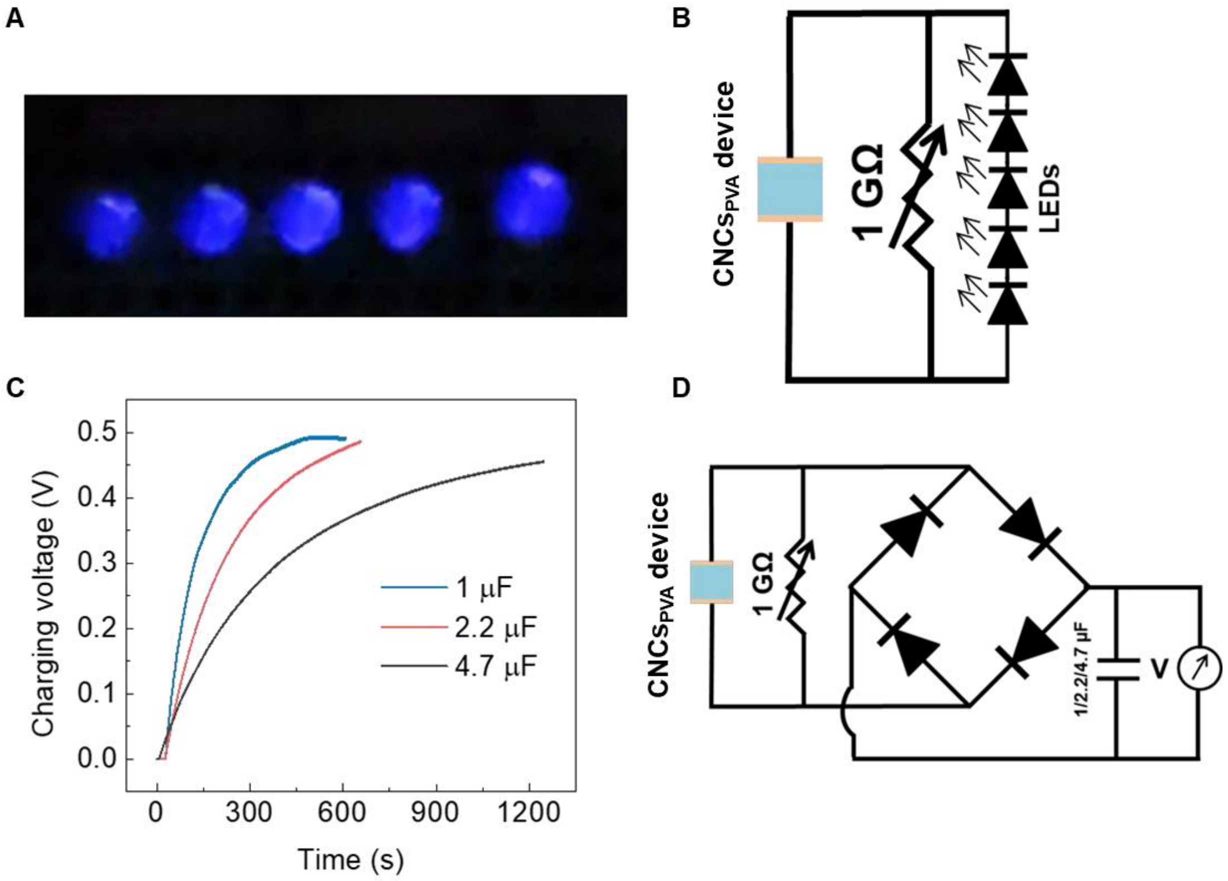

Fig. S17. Biodegradable piezoelectric devices can be used to pilot electronic components. A) Photograph of an array of 5 LEDs under operation, powered by a *NP*-1 CNCs$_{PVA}$ biodegradable device compressed by a repetitive pressure of 23 kPa. B) Scheme of the circuit used for driving the LEDs. C) Plot of capacitor charging, driven by a *NP*-1 CNCs$_{PVA}$ biodegradable device compressed by a repetitive pressure of 23 kPa. Different capacitance values are successfully tested: 1 μF, 2.2 μF and 4.7 μF. D) Scheme of the circuit used for charging the capacitor.

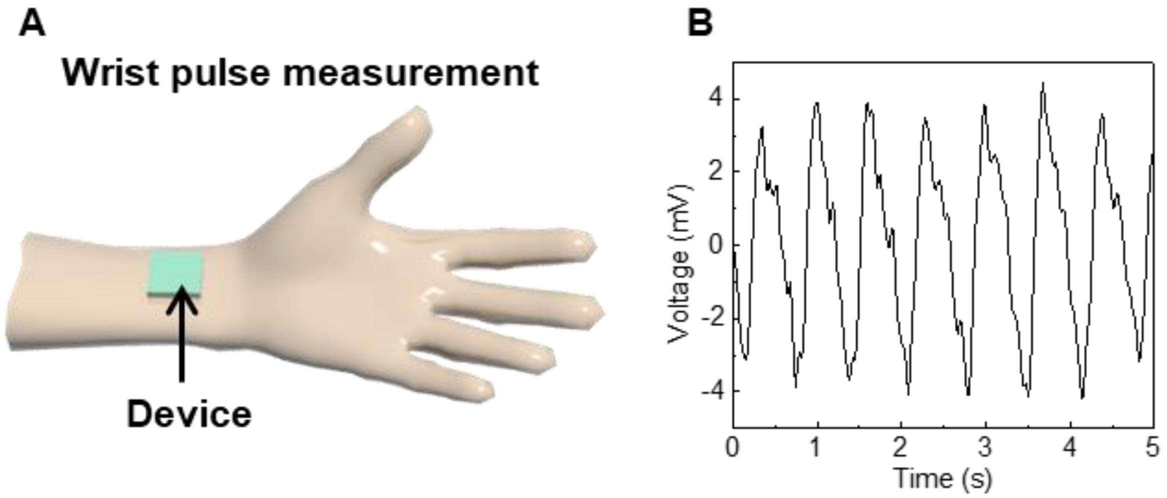

Fig. S18. Biodegradable piezoelectric devices can be used to detect the pressure of radial artery pulses. A) Schematics of the biodegradable device positioned on a human wrist to detect subtle pressure of radial artery pulses. B) Measured voltage signal *vs*. time (profile smoothed by using the Savitzky-Golay methods with 5 data points and ~1.15 Hz high-pass frequency filtering). Detected pulse rate: 84 min^{-1}.

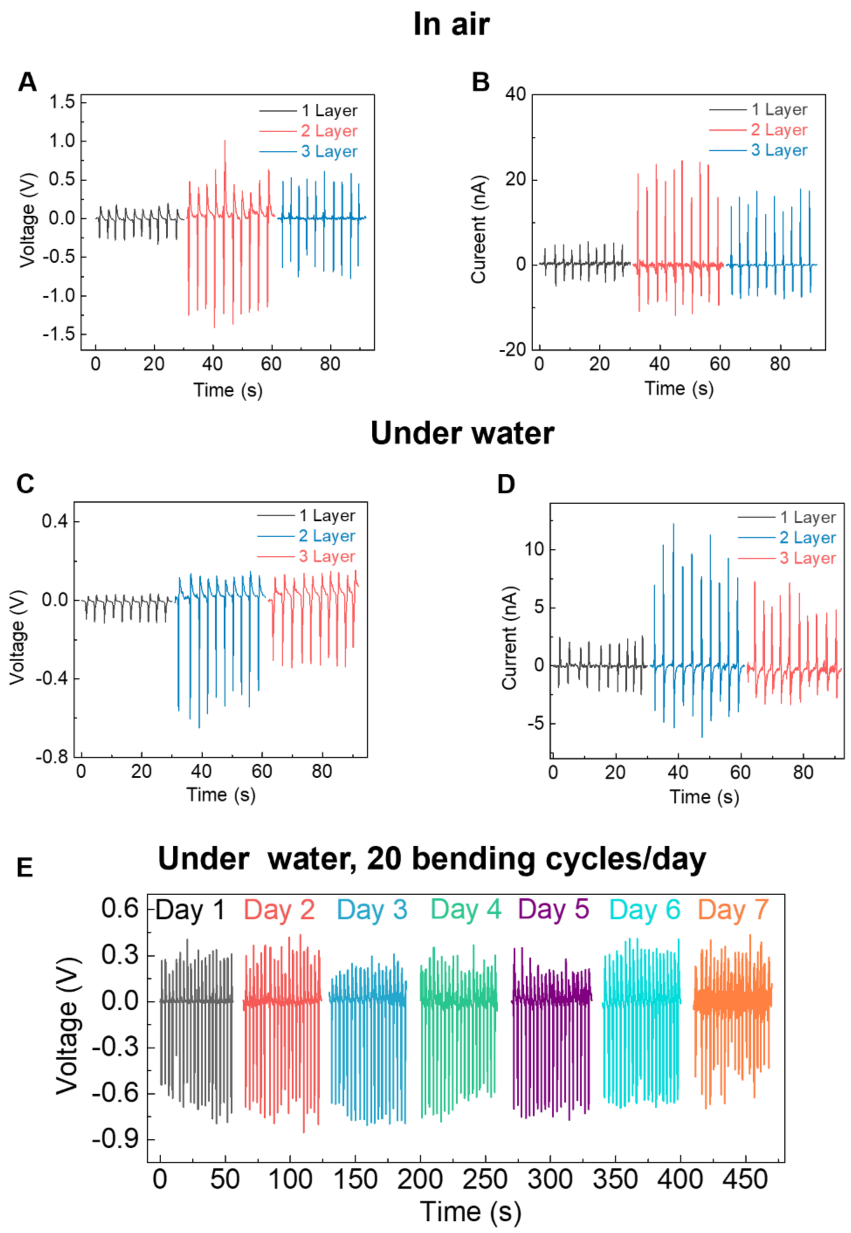

Fig. S19. Devices can work under cycling bending, in air as well as in liquid environment. Measured voltage (A,C) and current (B,D) output signals during bending cycles, in air (A,B) and under water (C,D). A strain of less than 1% is used. In air, maximum values for V_{oc} and I_{sc} are about 1.2 V and 18 nA, respectively. (E) Piezoelectric performance of the device in underwater environment. Measured voltage output signals during bending cycles, under water at temperature ~ 20-25 °C. Measurements are performed over a time interval of 7 days (20 bending cycles/day).

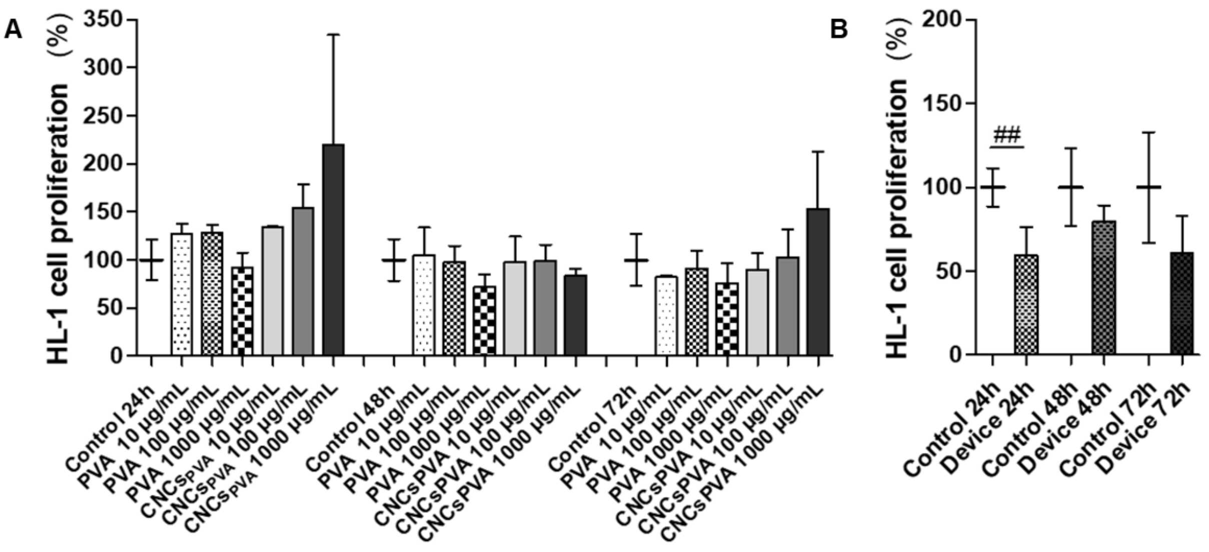

Fig. S20. The proliferation of cardiomyocyte cells is measured in presence of piezoelectric material. A) The proliferation of HL-1 cardiomyocyte cells cultured for 24, 48 and 72 hr with different amounts (10, 100, 1000 µg/mL) of PVA or $CNCs_{PVA}$ film dispersed in the culture medium. Results are reported in % over their untreated conditions (Control). One-way ANOVA, Dunnett's test. B) The proliferation of HL-1 cardiomyocyte cells cultured for 24, 48 and 72 on top of the biodegradable device. Results are reported in % over their untreated conditions (Control). ## $P < 0.01$, Student t-test. Data = mean ± SEM, *n*= 3; only Controls are reported as mean ± SD, to show the intra-assay variability.

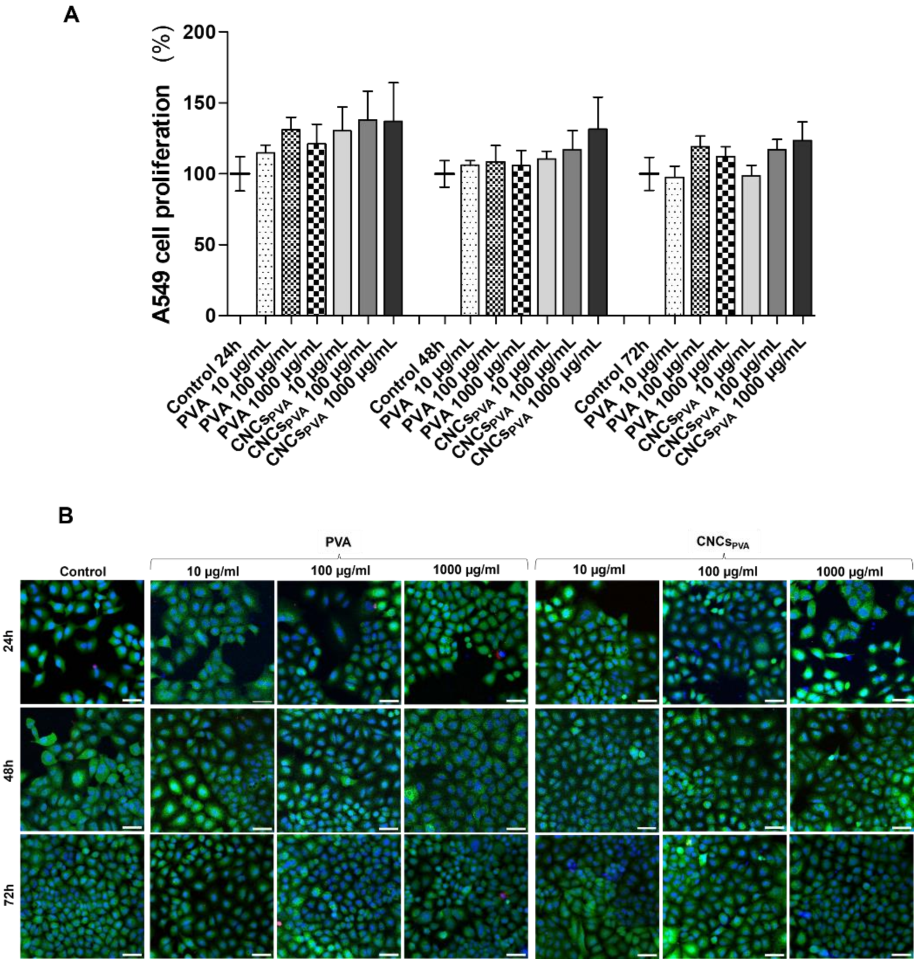

Fig. S21. The proliferation of lung epithelial cells is measured in presence of piezoelectric material. A) Proliferation of A549 lung epithelial cells cultured for 24, 48 and 72 h with different amounts (10, 100, 1000 µg/mL) of PVA or $CNCs_{PVA}$ dissolved from films in the culture medium. Results are reported as percentage with respect their untreated conditions (Control). One-way ANOVA, Dunnett's test. Data = mean ± standard error of the means, n= 3; only controls are reported as mean ± SD, to show the intra-assay variability. B) Vitality test: Fluorescence micrographs of A549 cells cultured with various amounts (10, 100, 1000 µg/mL) of PVA (left) and $CNCs_{PVA}$ (right) films dissolved in cell culture medium, during a period of 3 days of observation. A549 vital cells are visible in green (calcein-positive), all cell nuclei in blue (Hoechst33342-positive), and the level of necrotic/dying cells in red (PI-positive). Scale bar: 50 µm.

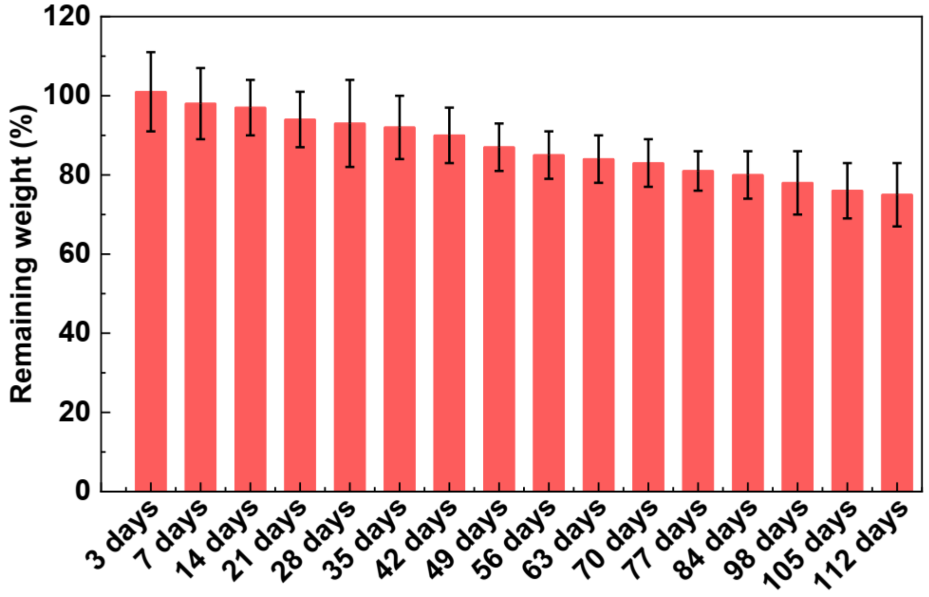

Fig. S22. Bioresorbability test are performed at 37°C in PBS solution with magnetic stirring. The percentage of the remaining material is calculated as the ratio of the dried weight (measured after washing the device in distilled water to remove residual PBS) to the original one (before soaking in PBS).

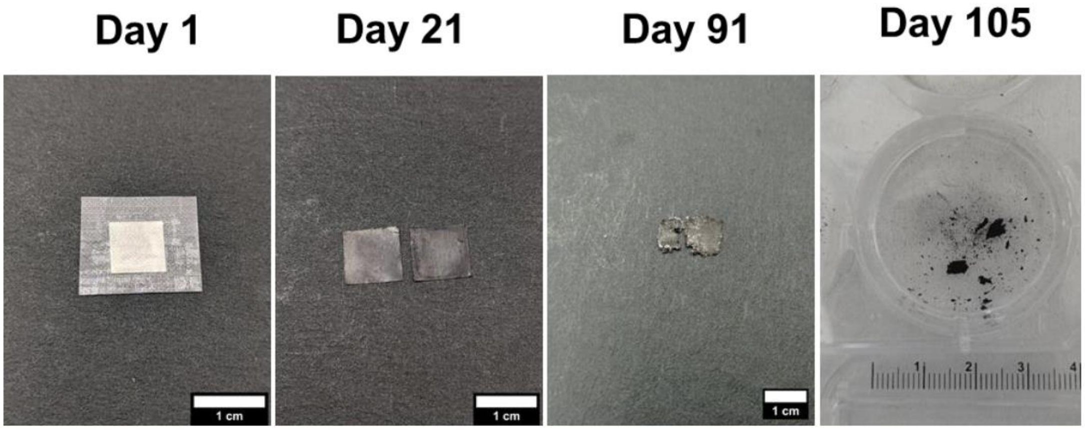

Fig. S23. Experiments showing accelerated degradation. Photographs showing a biodegradable device with a simplified design (small area and squared electrodes) at different days in the buffered solution at an accelerated-degradation temperature of 74 °C.

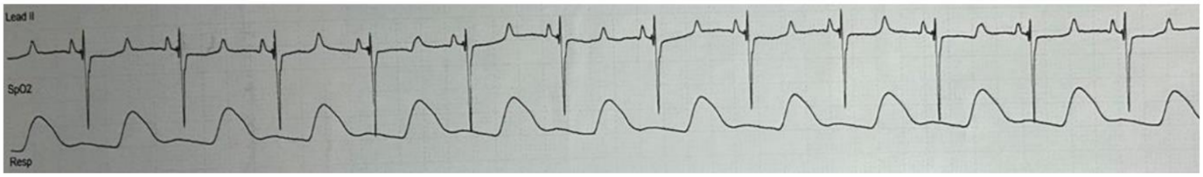

Fig. S24. Electrocardiogram (ECG) profiles are collected for swine embedded with the device. ECG and SpO2 signals recorded from pig with the biodegradable device sutured on heart.

Table S1. Pressure sensitivity of the $CNCs_{PVA}$ piezoelectric devices. V_{oc} and I_{sc} results are summarized for the different device configuration.

Device configuration	Voltage sensitivity (V/kPa)		Current sensitivity (nA/kPa)	
	Pressure range		Pressure range	
	0.76–4.55 kPa	6.8–37.3 kPa	0.76–4.55 kPa	6.8–37.3 kPa
Planar	0.21	0.09	4.6	1.0
NL-1	0.33	0.18	25.8	4.8
NL-2	0.37	0.15	22.6	2.3
***NP*-1**	**4.2**	**0.23**	**155.3**	**6.4**
NP-2	0.82	0.15	78.7	5.5

Table S2. ***NP*-1 CNCsPVA system exhibits high performance.** Comparison of the pressure sensitivity of the *NP*-1 $CNCs_{PVA}$ device encapsulated with PDMS and state-of-the-art piezoelectric and triboelectric devices.

		Material	Sensitivity (V/kPa)	Pressure range (kPa)	Ref.
**Piezoelectric devices**	Biodegradable	***NP*-1 CNCsPVA**	**4.2**	**0.7-5**	**This work**
			**0.23**	**7-37**	
		Biodegradable β-glycine/chitosan	4.2×10^{-3}	5−60	[44]
		Fish skin based nanogenerator	3.7×10^{-3}	<54	[45]
		PLLA/Glycine composite film	0.022	0-10	[46]
		Bacterial cellulose (BC) hydrogel/ imidazolium perchlorate (ImClO4) molecular ferroelectric	4.24×10^{-3}	0.2-31.25	[47]
		Microstructure Gelatine film	0.026	0.1-100	[48]
		Cellulose nanocrystals based 3-D printed nanogenerator	0.8	1-10	[49]
		Electrospun gelatine nanofibre	0.8	< 10	[50]
	Organic non-biodegradable	P(VDF-TrFE) thin film	0.74×10^{-3}	8-30	[51]
		Electrospun PVDF fabric	0.83×10^{-3}	150−250	[52]
		Pt-PVDF aligned nanofibres	1.6×10^{-3}	< 435	[53]
		P(VDF-TrFE)/$BaTiO_3$	2.615×10^{-3}	100−500	[54]
		PVDF/$BaTiO_3$ nanocomposite fibres	0.017	1−40	[55]
		Vertically integrated P(VDF-TrFE) fibre array	0.027	40−800	[56]

		3D printed P(VDF-TrFE) film	1.47	0.3–50	[57]
		PVDF/ZnO film	0.012	10-50	[58]
		P(VDF-TrFE)/PEDOT-PSS composite film	2.2	0.025–100	[59]
	Inorganic non-biodegradable	$Pb(Zr_{0.52}Ti_{0.48})O_3$ thin film on glass fiber fabric	60×10^{-6}	(120-480) $\times10^3$	[60]
		$BaTiO_3$ nanopillar array	0.025	50-300	[61]
		Br doped 2D ZnO nanosheets	0.086	0-300	[62]
		(PZT) single-crystal nanowires	0.14	15-70	[63]
		Sm-doped $Pb(Mg_{1/3}Nb_{2/3})O_3$-$PbTiO_3$ film	0.29	3.5-30	[64]
**Triboelectric devices**		PDMS, ITO	2.82×10^{-3}	<428.8	[65]
		Micro-frustum-array polydimethylsiloxane (mf-PDMS) film	0.567	0-600	[66]
		Self-polarized PVDF-TrFE, PDMS	0.104	0.05–5	[67]
		Latex membrane, FEP	0.44	2.3-3.2	[68]
		PDMS, PDMS/CNT nanocomposites	0.51	5-450	[69]
		Hierarchical PDMS, P(VDF-TrFE)	0.55	<20	[70]
		Multilayered PVDF-TrFE/BTO, Al	0.94	0.098–9.8	[71]
		PTFE, ITO	1.75	10–40	[72]

Table S3. *NP*-1 CNCsPVA system generates high piezoelectric output compared with other biomaterials. Comparison of the piezoelectric output performances in terms of the output voltage, current and power, of the *NP*-1 $CNCs_{PVA}$ biodegradable device with previously reported biomaterial-based platforms.

Material	Biodegradable Device platform (Yes/No)	Output voltage (V)	Output current (nA)	Output power density ($\mu W\ cm^{-2}$)	References
***NP*-1 $CNCs_{PVA}$**	**Yes**	**9**<br>**across 1 Gohm under 23 kPa (~ 2.3N)**	**400**<br>**across 1 Gohm under 23 kPa (~ 2.3N)**	**0.6**<br>**across 500 Mohms under 23 kPa (~ 2.3N)**	**This work**
PVA-cellulose electrospun nanofiber	No	0.04<br>under 14 N	300<br>under 14 N	1.1×10^{-2}	[73]
Cellulose nanofiber film	No	0.1	//	//	[29]
Bacterial cellulose hydrogel/ imidazolium perchlorate ($ImClO_4$) molecular ferroelectric	No	0.14<br>under 31.25 kPa	//	//	[47]
PVA/DL-alanine composite film	No	0.15	//	//	[74]
Fish skin based collagen	No	0.2<br>across 35 Mohms<br>under 7.5 N	2<br>across 35 Mohms under 7.5 N	4.6×10^{-3}<br>across 35 Mohms under 7.5 N	[45]
M13 bacteriophage nanopillars	No	0.232<br>across 1 Mohm under 30 N	11.1<br>across 1 Mohm under 30 N	0.29×10^{-3}<br>across 10 Mohms under 30 N	[75]
M13 bacteriophage (phage)	No	0.4<br>under 34 N	4<br>under 34 N	//	[4]

PLLA/glycine composite film	No	0.42 under 69.5 kPa	~170 nA across 400 k ohms resistance	4.17 $\times 10^{-6}$ across 400 kohms resistance	[46]
γ-glycine	No	0.45 under 0.172 N	//	//	[5]
Delignifed wood sponge	Yes	0.69 under 13.3 kPa	7.1 under 13.3 kPa	0.6 $\times 10^{-3}$ across 80 Mohms resistance, under 13.3 kPa	[76]
Decayed wood sponge	Yes	1.7 under 45 kPa	26 under 45 kPa	//	[77]
2D-dot patterns M13 phage	No	0.95 under ~65 N	94 under ~65 N	80×10^{-3} under ~65 N	[78]
Cycloglycine - tryptophan	No	1.2 under 65 N	1.75 under 65 N	//	[79]
PLLA stretched film	Yes	1.3 under 17 N	300 under 14 N	//	[34]
Diphenylalan in e (FF) peptide microrod	No	1.4 under 60 N	39.2 under 60 N	3.3 $\times 10^{-3}$ across 50 Mohms under 60 N	[80]
PLLA nanofiber	Yes	1.5 under 60 kPa	//	//	[81]
PVA/Glycine composite film	Yes	2 under 30 N	180 under 30 N	//	[13]
Microstructur e Gelatine film	Yes	2.3 under 113 kPa	8.9 under 113 kPa	0.3 across 8 Mohms under 113 kPa	[48]

Diphenylalanine (FF) peptide nanotubes	No	2.8 across 1 Gohm under 42 N	37.4 under 42 N	//	[82]
Vertically aligned M13 phage nanofibres	No	2.8 across 1 Gohm under 17 N	120 under 17 N	//	[83]
Electrospun silk nanofiber membrane	No	6 across 100 Mohms under 8 kPa	150 under 8kPa	//	[84]

Movie S1. This video demonstrated the robustness of S-IL patterned *NP*-1 $CNCs_{PVA}$ on Mo electrodes after repetitive compression of 23 kPa pressure at 1 Hz for the duration of about 15 minutes/days for 7 days.

Movie S2. This video demonstrated the real-time measurement of radial artery pulses by positioning the biodegradable device on a human wrist.

REFERENCES AND NOTES

1. E. Fukada, Piezoelectricity of wood. *J. Physical Soc. Japan* **10**, 149–154 (1955).

2. E. Fukada, I. Yasuda, On the piezoelectric effect of bone. *J. Physical Soc. Japan* **12**, 1158–1162 (1957).

3. M. Minary-Jolandan, M.-F. Yu, Shear piezoelectricity in bone at the nanoscale. *Appl. Phys. Lett.* **97**, 153127 (2010).

4. B. Y. Lee, J. Zhang, C. Zueger, W.-J. Chung, S. Y. Yoo, E. Wang, J. Meyer, R. Ramesh, S.-W. Lee, Virus-based piezoelectric energy generation. *Nat. Nanotechnol.* **7**, 351–356 (2012).

5. S. Guerin, A. Stapleton, D. Chovan, R. Mouras, M. Gleeson, C. McKeown, M. R. Noor, C. Silien, F. M. Rhen, A. L. Kholkin, N. Liu, T. Soulimane, S. A. M. Tofail, D. Thompson, Control of piezoelectricity in amino acids by supramolecular packing. *Nat. Mater.* **17**, 180–186 (2018).

6. R. Lay, G. S. Deijs, J. Malmström, The intrinsic piezoelectric properties of materials–A review with a focus on biological materials. *RSC Adv.* **11**, 30657–30673 (2021).

7. T. D. Nguyen, N. Deshmukh, J. M. Nagarah, T. Kramer, P. K. Purohit, M. J. Berry, M. C. McAlpine, Piezoelectric nanoribbons for monitoring cellular deformations. *Nat. Nanotechnol.* **7**, 587–593 (2012).

8. C. Dagdeviren, P. Joe, O. L. Tuzman, K.-I. Park, K. J. Lee, Y. Shi, Y. Huang, J. A. Rogers, Recent progress in flexible and stretchable piezoelectric devices for mechanical energy harvesting, sensing and actuation. *Extreme Mech. Lett.* **9**, 269–281 (2016).

9. X. Wan, Z. Wang, X. Zhao, Q. Hu, Z. Li, Z. L. Wang, L. Li, Flexible and highly piezoelectric nanofibers with organic–inorganic coaxial structure for self-powered physiological multimodal sensing. *Chem. Eng. J.* **451**, 139077 (2023).

10. S. Min, D. H. Kim, D. J. Joe, B. W. Kim, Y. H. Jung, J. H. Lee, B. Y. Lee, I. Doh, J. An, Y. N. Youn, B. Joung, C. D. Yoo, H.-S. Ahn, K. J. Lee, Clinical validation of a wearable

piezoelectric blood-pressure sensor for continuous health monitoring. *Adv. Mater.* **35**, 2301627 (2023).

11. T. Yucel, P. Cebe, D. L. Kaplan, Structural origins of silk piezoelectricity. *Adv. Funct. Mater.* **21**, 779–785 (2011).

12. J. Wang, C. Carlos, Z. Zhang, J. Li, Y. Long, F. Yang, Y. Dong, X. Qiu, Y. Qian, X. Wang, Piezoelectric nanocellulose thin film with large-scale vertical crystal alignment. *ACS Appl. Mater. Interfaces* **12**, 26399–26404 (2020).

13. F. Yang, J. Li, Y. Long, Z. Zhang, L. Wang, J. Sui, Y. Dong, Y. Wang, R. Taylor, D. Ni, W. Cai, P. Wang, T. Hacker, X. Wang, Wafer-scale heterostructured piezoelectric bio-organic thin films. *Science* **373**, 337–342 (2021).

14. R. Wang, J. Sui, X. Wang, Natural piezoelectric biomaterials: A biocompatible and sustainable building block for biomedical devices. *ACS Nano* **16**, 17708–17728 (2022).

15. D. Klemm, B. Heublein, H. P. Fink, A. Bohn, Cellulose: Fascinating biopolymer and sustainable raw material. *Angew. Chem. Int. Ed.* **44**, 3358–3393 (2005).

16. Y. Song, Z. Shi, G.-H. Hu, C. Xiong, A. Isogai, Q. Yang, Recent advances in cellulose-based piezoelectric and triboelectric nanogenerators for energy harvesting: A review. *J. Mater. Chem. A* **9**, 1910–1937 (2021).

17. D. Klemm, F. Kramer, S. Moritz, T. Lindström, M. Ankerfors, D. Gray, A. Dorris, Nanocelluloses: A new family of nature-based materials. *Angew. Chem. Int. Ed.* **50**, 5438–5466 (2011).

18. Y. García, Y. B. Ruiz-Blanco, Y. Marrero-Ponce, C. M. Sotomayor-Torres, Orthotropic piezoelectricity in 2D nanocellulose. *Sci. Rep.* **6**, 34616 (2016).

19. R. J. Moon, A. Martini, J. Nairn, J. Simonsen, J. Youngblood, Cellulose nanomaterials review: Structure, properties and nanocomposites. *Chem. Soc. Rev.* **40**, 3941–3994 (2011).

20. K. A. Werling, G. R. Hutchison, D. S. Lambrecht, Piezoelectric effects of applied electric fields on hydrogen-bond interactions: First-principles electronic structure investigation of weak electrostatic interactions. *J. Phys. Chem. Lett.* **4**, 1365–1370 (2013).

21. I. Chae, C. K. Jeong, Z. Ounaies, S. H. Kim, Review on electromechanical coupling properties of biomaterials. *ACS Appl. Bio Mater.* **1**, 936–953 (2018).

22. L. Csoka, I. C. Hoeger, O. J. Rojas, I. Peszlen, J. J. Pawlak, P. N. Peralta, Piezoelectric effect of cellulose nanocrystals thin films. *ACS Macro Lett.* **1**, 867–870 (2012).

23. B. Frka-Petesic, B. Jean, L. Heux, First experimental evidence of a giant permanent electric-dipole moment in cellulose nanocrystals. *EPL* **107**, 28006 (2014).

24. L. Zhai, H. C. Kim, J. W. Kim, J. Kim, Alignment effect on the piezoelectric properties of ultrathin cellulose nanofiber films. *ACS Appl. Bio Mater.* **3**, 4329–4334 (2020).

25. J. R. Capadona, K. Shanmuganathan, D. J. Tyler, S. J. Rowan, C. Weder, Stimuli-responsive polymer nanocomposites inspired by the sea cucumber dermis. *Science* **319**, 1370–1374 (2008).

26. C. Miao, D. Mauran, W. Y. Hamad, How hydrogen-bonding interactions and nanocrystal aspect ratios influence the morphology and mechanical performance of polymer nanocomposites reinforced with cellulose nanocrystals. *Soft Matter* **18**, 4572–4581 (2022).

27. J. Han, C. Zhou, Y. Wu, F. Liu, Q. Wu, Self-assembling behavior of cellulose nanoparticles during freeze-drying: Effect of suspension concentration, particle size, crystal structure, and surface charge. *Biomacromolecules* **14**, 1529–1540 (2013).

28. O. N. Tretinnikov, S. A. Zagorskaya, Determination of the degree of crystallinity of poly (vinyl alcohol) by FTIR spectroscopy. *J. Appl. Spectrosc* **79**, 521–526 (2012).

29. A. Sultana, M. M. Alam, E. Pavlopoulou, E. Solano, M. Berggren, X. Crispin, D. Zhao, Toward high-performance green piezoelectric generators based on electrochemically poled nanocellulose. *Chem. Mater.* **35**, 1568–1578 (2023).

30. Y.-M. You, W.-Q. Liao, D. Zhao, H.-Y. Ye, Y. Zhang, Q. Zhou, X. Niu, J. Wang, P.-F. Li, D.-W. Fu, Z. Wang, S. Gao, K. Yang, J.-M. Liu, J. Li, Y. Yan, R.-G. Xiong, An organic-inorganic perovskite ferroelectric with large piezoelectric response. *Science* **357**, 306–309 (2017).

31. V. Bhavanasi, D. Y. Kusuma, P. S. Lee, Polarization orientation, piezoelectricity, and energy harvesting performance of ferroelectric PVDF-TrFE nanotubes synthesized by nanoconfinement. *Adv. Energy Mater.* **4**, 1400723 (2014).

32. S. Cha, S. M. Kim, H. Kim, J. Ku, J. I. Sohn, Y. J. Park, B. G. Song, M. H. Jung, E. K. Lee, B. L. Choi, J. J. Park, Z. L. Wang, J. M. Kim, K. Kim, Porous PVDF as effective sonic wave driven nanogenerators. *Nano Lett.* **11**, 5142–5147 (2011).

33. J. Zhang, S. Ye, H. Liu, X. Chen, X. Chen, B. Li, W. Tang, Q. Meng, P. Ding, H. Tian, X. Li, Y. Zhang, P. Xu, J. Shao, 3D printed piezoelectric BNNTs nanocomposites with tunable interface and microarchitectures for self-powered conformal sensors. *Nano Energy* **77**, 105300 (2020).

34. E. J. Curry, K. Ke, M. T. Chorsi, K. S. Wrobel, A. N. Miller, A. Patel, I. Kim, J. Feng, L. Yue, Q. Wu, C.-L. Kuo, K. W.-H. Lo, C. T. Laurencin, H. Ilies, P. K. Purohit, T. D. Nguyen, Biodegradable piezoelectric force sensor. *Proc. Natl. Acad. Sci. U.S.A.* **115**, 909–914 (2018).

35. M. Sikora-Jasinska, L. M. Morath, M. P. Kwesiga, M. E. Plank, A. L. Nelson, A. A. Oliver, M. L. Bocks, R. J. Guillory II, J. Goldman, In-vivo evaluation of molybdenum as bioabsorbable stent candidate. *Bioact. Mater.* **14**, 262–271 (2022).

36. R. Das, E. J. Curry, T. T. Le, G. Awale, Y. Liu, S. Li, J. Contreras, C. Bednarz, J. Millender, X. Xin, D. Rowe, S. Emadi, K. W. H. Lo, T. D. Nguyen, Biodegradable nanofiber bone-tissue scaffold as remotely-controlled and self-powering electrical stimulator. *Nano Energy* **76**, 105028 (2020).

37. T. Vinikoor, G. K. Dzidotor, T. T. Le, Y. Liu, H.-M. Kan, S. Barui, M. T. Chorsi, E. J. Curry, E. Reinhardt, H. Wang, P. Singh, M. A. Merriman, E. D'Orio, J. Park, S. Xiao, J. H. Chapman, F. Lin, C.-S. Truong, S. Prasadh, L. Chuba, S. Killoh, S.-W. Lee, Q. Wu, R. M.

Chidambaram, K. W. H. Lo, C. T. Laurencin, T. D. Nguyen, Injectable and biodegradable piezoelectric hydrogel for osteoarthritis treatment. *Nat. Commun.* **14**, 6257 (2023).

38. W. Pi, F. Rao, J. Cao, M. Zhang, T. Chang, Y. Han, Y. Zheng, S. Liu, Q. Li, X. Sun, Y. Shao, Sono-electro-mechanical therapy for peripheral nerve regeneration through piezoelectric nanotracts. *Nano Today* **50**, 101860 (2023).

39. M. T. Chorsi, T. T. Le, F. Lin, T. Vinikoor, R. Das, J. F. Stevens, C. Mundrane, J. Park, K. T. M. Tran, Y. Liu, J. Pfund, R. Thompson, W. He, M. Jain, M. D. Morales-Acosta, O. R. Bilal, K. Kazerounian, H. Ilies, T. D. Nguyen, Highly piezoelectric, biodegradable, and flexible amino acid nanofibers for medical applications. *Sci. Adv.* **9**, eadg6075 (2023).

40. L. Zhang, C. Marcus, D. Lin, D. Mejorado, S. J. Schoen, T. T. Pierce, V. Kumar, S. V. Fernandez, D. Hunt, Q. Li, I. I. Shuvo, D. Sadat, W. Du, H. Edenbaum, L. Jin, W. Liu, Y. C. Eldar, F. Li, A. P. Chandrakasan, A. E. Samir, C. Dagdeviren, A conformable phased-array ultrasound patch for bladder volume monitoring. *Nat. Electron.* **7**, 77–90 (2024).

41. W. C. Claycomb, N. A. Lanson Jr., B. S. Stallworth, D. B. Egeland, J. B. Delcarpio, A. Bahinski, N. J. Izzo Jr., HL-1 cells: A cardiac muscle cell line that contracts and retains phenotypic characteristics of the adult cardiomyocyte. *Proc. Natl. Acad. Sci. U.S.A.* **95**, 2979–2984 (1998).

42. M. Gagliardi, S. Chiarugi, C. De Cesari, G. Di Gregorio, A. Diodati, L. Baroncelli, M. Cecchini, I. Tonazzini, Crosslinked chitosan nanoparticles with muco-adhesive potential for intranasal delivery applications. *Int. J. Mol. Sci.* **24**, 6590 (2023).

43. I. Tonazzini, C. Masciullo, E. Savi, A. Sonato, F. Romanato, M. Cecchini, Neuronal contact guidance and YAP signaling on ultra-small nanogratings. *Sci. Rep.* **10**, 3742 (2020).

44. E. S. Hosseini, L. Manjakkal, D. Shakthivel, R. Dahiya, Glycine–chitosan-based flexible biodegradable piezoelectric pressure sensor. *ACS Appl. Mater. Interfaces* **12**, 9008–9016 (2020).

45. S. K. Ghosh, D. Mandal, Sustainable energy generation from piezoelectric biomaterial for noninvasive physiological signal monitoring. *ACS Sustain. Chem. Eng.* **5**, 8836–8843 (2017).

46. M. Ali, S. M. Hoseyni, R. Das, M. Awais, I. Basdogan, L. Beker, A Flexible and biodegradable piezoelectric-based wearable sensor for non-invasive monitoring of dynamic human motions and physiological signals. *Adv. Mater. Technol.* **8**, 2300347 (2023).

47. J. Lu, S. Hu, W. Li, X. Wang, X. Mo, X. Gong, H. Liu, W. Luo, W. Dong, C. Sima, Y. Wang, G. Yang, J.-T. Luo, S. Jiang, Z. Shi, G. Zhang, A biodegradable and recyclable piezoelectric sensor based on a molecular ferroelectric embedded in a bacterial cellulose hydrogel. *ACS Nano* **16**, 3744–3755 (2022).

48. S. K. Ghosh, J. Park, S. Na, M. P. Kim, H. Ko, A fully biodegradable ferroelectric skin sensor from edible porcine skin gelatine. *Adv. Sci.* **8**, 2005010 (2021).

49. K. Maity, A. Mondal, M. C. Saha, Cellulose nanocrystal-based all-3D-printed pyro-piezoelectric nanogenerator for hybrid energy harvesting and self-powered cardiorespiratory monitoring toward the human–machine interface. *ACS Appl. Mater. Interfaces* **15**, 13956–13970 (2023).

50. S. K. Ghosh, P. Adhikary, S. Jana, A. Biswas, V. Sencadas, S. D. Gupta, B. Tudu, D. Mandal, Electrospun gelatin nanofiber based self-powered bio-e-skin for health care monitoring. *Nano Energy* **36**, 166–175 (2017).

51. T. Sharma, K. Aroom, S. Naik, B. Gill, J. X. J. Zhang, Flexible thin-film PVDF-TrFE based pressure sensor for smart catheter applications. *Ann. Biomed. Eng.* **41**, 744–751 (2013).

52. Y. R. Wang, J. M. Zheng, G. Y. Ren, P. H. Zhang, C. Xu, A flexible piezoelectric force sensor based on PVDF fabrics. *Smart Mater. Struct.* **20**, 045009 (2011).

53. S. K. Ghosh, D. Mandal, Synergistically enhanced piezoelectric output in highly aligned 1D polymer nanofibers integrated all-fiber nanogenerator for wearable nano-tactile sensor. *Nano Energy* **53**, 245–257 (2018).

54. N. T. Tien, S. Jeon, D.-I. Kim, T. Q. Trung, M. Jang, B.-U. Hwang, K.-E. Byun, J. Bae, E. Lee, J. B.-H. Tok, Z. Bao, N.-E. Lee, J.-J. Park, A flexible bimodal sensor array for simultaneous sensing of pressure and temperature. *Adv. Mater.* **26**, 796–804 (2014).

55. W. Guo, C. Tan, K. Shi, J. Li, X.-X. Wang, B. Sun, X. Huang, Y.-Z. Long, P. Jiang, Wireless piezoelectric devices based on electrospun PVDF/$BaTiO_3$ NW nanocomposite fibers for human motion monitoring. *Nanoscale* **10**, 17751–17760 (2018).

56. X. Chen, H. Tian, X. Li, J. Shao, Y. Ding, N. An, Y. Zhou, A high performance P(VDF-TrFE) nanogenerator with self-connected and vertically integrated fibers by patterned EHD pulling. *Nanoscale* **7**, 11536–11544 (2015).

57. X. Yuan, X. Gao, X. Shen, J. Yang, Z. Li, S. Dong, A 3D-printed, alternatively tilt-polarized PVDF-TrFE polymer with enhanced piezoelectric effect for self-powered sensor application. *Nano Energy* **85**, 105985 (2021).

58. M. Yuan, R. Ma, Q. Ye, X. Bai, H. Li, F. Yan, C. Liu, Y. Ren, Z. Wang, Melt-stretched poly(vinylidene fluoride)/zinc oxide nanocomposite films with enhanced piezoelectricity by stress concentrations in piezoelectric domains for wearable electronics. *Chem. Eng. J.* **455**, 140771 (2023).

59. B. Li, C. Cai, Y. Liu, F. Wang, B. Yang, Q. Li, P. Zhang, B. Deng, P. Hou, W. Liu, Ultrasensitive mechanical/thermal response of a P(VDF-TrFE) sensor with a tailored network interconnection interface. *Nat. Commun.* **14**, 4000 (2023).

60. S. He, W. Dong, Y. Guo, L. Guan, H. Xiao, H. Liu, Piezoelectric thin film on glass fiber fabric with structural hierarchy: An approach to high-performance, superflexible, cost-effective, and large-scale nanogenerators. *Nano Energy* **59**, 745–753 (2019).

61. S.-H. Shin, S.-Y. Choi, M. H. Lee, J. Nah, High-performance piezoelectric nanogenerators via imprinted sol–gel $BaTiO_3$ nanopillar array. *ACS Appl. Mater. Interfaces* **9**, 41099–41103 (2017).

62. S. Rafique, A. K. Kasi, A. , J. K. Kasi, M. Bokhari, Z. Shakoor, Fabrication of Br doped ZnO nanosheets piezoelectric nanogenerator for pressure and position sensing applications. *Curr. Appl. Phys.* **21**, 72–79 (2021).

63. Q.-L. Zhao, G.-P. He, J.-J. Di, W.-L. Song, Z.-L. Hou, P.-P. Tan, D.-W. Wang, M.-S. Cao, Flexible semitransparent energy harvester with high pressure sensitivity and power density based on laterally aligned PZT single-crystal nanowires. *ACS Appl. Mater. Interfaces* **9**, 24696–24703 (2017).

64. P. Lv, J. Qian, C. Yang, T. Liu, Y. Wang, D. Wang, S. Huang, X. Cheng, Z. Cheng, Flexible all-inorganic Sm-doped PMN-PT film with ultrahigh piezoelectric coefficient for mechanical energy harvesting, motion sensing, and human-machine interaction. *Nano Energy* **97**, 107182 (2022).

65. X.-Z. Jiang, Y.-J. Sun, Z. Fan, T.-Y. Zhang, Integrated flexible, waterproof, transparent, and self-powered tactile sensing panel. *ACS Nano* **10**, 7696–7704 (2016).

66. J. Yu, X. Hou, J. He, M. Cui, C. Wang, W. Geng, J. Mu, B. Han, X. Chou, Ultra-flexible and high-sensitive triboelectric nanogenerator as electronic skin for self-powered human physiological signal monitoring. *Nano Energy* **69**, 104437 (2020).

67. K. Parida, V. Bhavanasi, V. Kumar, R. Bendi, P. S. Lee, Self-powered pressure sensor for ultra-wide range pressure detection. *Nano Research* **10**, 3557–3570 (2017).

68. P. Bai, G. Zhu, Q. Jing, J. Yang, J. Chen, Y. Su, J. Ma, G. Zhang, Z. L. Wang, Membrane-based self-powered triboelectric sensors for pressure change detection and its uses in security surveillance and healthcare monitoring. *Adv. Funct. Mater.* **24**, 5807–5813 (2014).

69. M. S. Rasel, P. Maharjan, M. Salauddin, M. T. Rahman, H. O. Cho, J. W. Kim, J. Y. Park, An impedance tunable and highly efficient triboelectric nanogenerator for large-scale, ultra-sensitive pressure sensing applications. *Nano Energy* **49**, 603–613 (2018).

70. M. Ha, S. Lim, S. Cho, Y. Lee, S. Na, C. Baig, H. Ko, Skin-inspired hierarchical polymer architectures with gradient stiffness for spacer-free, ultrathin, and highly sensitive triboelectric sensors. *ACS Nano* **12**, 3964–3974 (2018).

71. Y. Park, Y.-E. Shin, J. Park, Y. Lee, M. P. Kim, Y.-R. Kim, S. Na, S. K. Ghosh, H. Ko, Ferroelectric multilayer nanocomposites with polarization and stress concentration structures for enhanced triboelectric performances. *ACS Nano* **14**, 7101–7110 (2020).

72. J. Zou, M. Zhang, J. Huang, J. Bian, Y. Jie, M. Willander, X. Cao, N. Wang, Z. L. Wang, Coupled supercapacitor and triboelectric nanogenerator boost biomimetic pressure sensor. *Adv. Energy Mater.* **8**, 1702671 (2018).

73. E. S. Choi, H. C. Kim, R. M. Muthoka, P. S. Panicker, D. O. Agumba, J. Kim, Aligned cellulose nanofiber composite made with electrospinning of cellulose nanofiber – Polyvinyl alcohol and its vibration energy harvesting. *Compos. Sci. Technol.* **209**, 108795 (2021).

74. B. Jeon, D. Han, G. Yoon, Piezoelectric characteristics of PVA/DL-alanine polycrystals in d_{33} mode. *iScience* **26**, 105768 (2023).

75. D.-M. Shin, H. J. Han, W.-G. Kim, E. Kim, C. Kim, S. W. Hong, H. K. Kim, J.-W. Oh, Y.-H. Hwang, Bioinspired piezoelectric nanogenerators based on vertically aligned phage nanopillars. *Energ. Environ. Sci.* **8**, 3198–3203 (2015).

76. J. Sun, H. Guo, J. Ribera, C. Wu, K. Tu, M. Binelli, G. Panzarasa, F. W. M. R. Schwarze, Z. L. Wang, I. Burgert, Sustainable and biodegradable wood sponge piezoelectric nanogenerator for sensing and energy harvesting applications. *ACS Nano* **14**, 14665–14674 (2020).

77. J. Sun, H. Guo, G. N. Schädli, K. Tu, S. Schär, F. W. M. R. Schwarze, G. Panzarasa, J. Ribera, I. Burgert, Enhanced mechanical energy conversion with selectively decayed wood. *Sci. Adv.* **7**, eabd9138 (2021).

78. K. Heo, H.-E. Jin, H. Kim, J. H. Lee, E. Wang, S.-W. Lee, Transient self-templating assembly of M13 bacteriophage for enhanced biopiezoelectric devices. *Nano Energy* **56**, 716–723 (2019).

79. K. Tao, W. Hu, B. Xue, D. Chovan, N. Brown, L. J. W. Shimon, O. Maraba, Y. Cao, S. A. M. Tofail, D. Thompson, J. Li, R. Yang, E. Gazit, Bioinspired stable and photoluminescent assemblies for power generation. *Adv. Mater.* **31**, e1807481 (2019).

80. V. Nguyen, R. Zhu, K. Jenkins, R. Yang, Self-assembly of diphenylalanine peptide with controlled polarization for power generation. *Nat. Commun.* **7**, 13566 (2016).

81. E. J. Curry, T. T. Le, R. Das, K. Ke, E. M. Santorella, D. Paul, M. T. Chorsi, K. T. M. Tran, J. Baroody, E. R. Borges, B. Ko, A. Golabchi, X. Xin, D. Rowe, L. Yue, J. Feng, M. D. Morales-Acosta, Q. Wu, I.-P. Chen, X. T. Cui, J. Pachter, T. D. Nguyen, Biodegradable nanofiber-based piezoelectric transducer. *Proc. Natl. Acad. Sci. U.S.A.* **117**, 214–220 (2020).

82. J.-H. Lee, K. Heo, K. Schulz-Schönhagen, J. H. Lee, M. S. Desai, H.-E. Jin, S.-W. Lee, Diphenylalanine peptide nanotube energy harvesters. *ACS Nano* **12**, 8138–8144 (2018).

83. J.-H. Lee, J. H. Lee, J. Xiao, M. S. Desai, X. Zhang, S.-W. Lee, Vertical self-assembly of polarized phage nanostructure for energy harvesting. *Nano Lett.* **19**, 2661–2667 (2019).

84. C. Sohn, H. Kim, J. Han, K.-T. Lee, A. Šutka, C. K. Jeong, Generating electricity from molecular bonding-correlated piezoresponse of biodegradable silk nanofibers. *Nano Energy* **103**, 107844 (2022).
